# Ancient recombination events and the origins of hepatitis E virus

**DOI:** 10.1186/s12862-016-0785-y

**Published:** 2016-10-12

**Authors:** Andrew G. Kelly, Natalie E. Netzler, Peter A. White

**Affiliations:** School of Biotechnology and Biomolecular Sciences, Faculty of Science, University of New South Wales, Sydney, NSW Australia

**Keywords:** Hepatitis E virus, Hepatitis, Recombination, *Hepeviridae*, *Astroviridae*, Evolution

## Abstract

**Background:**

Hepatitis E virus (HEV) is an enteric, single-stranded, positive sense RNA virus and a significant etiological agent of hepatitis, causing sporadic infections and outbreaks globally. Tracing the evolutionary ancestry of HEV has proved difficult since its identification in 1992, it has been reclassified several times, and confusion remains surrounding its origins and ancestry.

**Results:**

To reveal close protein relatives of the *Hepeviridae* family, similarity searching of the GenBank database was carried out using a complete *Orthohepevirus* A, HEV genotype I (GI) ORF1 protein sequence and individual proteins. The closest non-*Hepeviridae* homologues to the HEV ORF1 encoded polyprotein were found to be those from the lepidopteran-infecting *Alphatetraviridae* family members. A consistent relationship to this was found using a phylogenetic approach; the *Hepeviridae* RdRp clustered with those of the *Alphatetraviridae* and *Benyviridae* families. This puts the *Hepeviridae* ORF1 region within the “Alpha-like” super-group of viruses. In marked contrast, the HEV GI capsid was found to be most closely related to the chicken astrovirus capsid, with phylogenetic trees clustering the *Hepeviridae* capsid together with those from the *Astroviridae* family, and surprisingly within the “Picorna-like” supergroup. These results indicate an ancient recombination event has occurred at the junction of the non-structural and structure encoding regions, which led to the emergence of the entire *Hepeviridae* family.

The *Astroviridae* capsid is also closely related to the *Tymoviridae* family of monopartite, T = 3 icosahedral plant viruses, whilst its non-structural region is related to viruses of the *Potyviridae*; a large family of plant-infecting viruses with a flexible filamentous rod-shaped virion. Thus, we identified a separate inter-viral family recombination event, again at the non-structural/structural junction, which likely led to the creation of the *Astroviridae*.

**Conclusions:**

In summary, we have shown that new viral families have been created though recombination at the junction of the genome that encodes non-structural and structural proteins, and such recombination events are implicated in the genesis of important human pathogens; HEV, astrovirus and rubella virus.

**Electronic supplementary material:**

The online version of this article (doi:10.1186/s12862-016-0785-y) contains supplementary material, which is available to authorized users.

## Background

Hepatitis E virus (HEV) is a single-stranded RNA, non-enveloped enteric virus and a major etiological agent responsible for acute hepatitis, causing sporadic infections and widespread epidemics globally. Based on seroprevalence, it is estimated that one third of the global population has been infected with HEV at some stage in their lifetime [1]. HEV creates a significant health burden on society, with annual estimates of 20 million clinical infections, over three million reported acute cases, 3,000 stillbirths and 50,000 HEV-related deaths globally [2, 3]. However, it is likely that these figures are an underestimation, given that the virus is often asymptomatic, usually self-limiting and HEV epidemics strike in geographic areas that are the least likely to have effective diagnostic testing available [4, 5].

The *Hepeviridae* family is currently divided into two genera, *Orthohepevirus* and *Piscihepevirus* [6]. These genera include the species: *Orthohepevirus* A, which infects humans, swine, boar, rabbits, camels, deer and mongooses, *Orthohepevirus* B, which infects birds, *Orthohepevirus* C, which infects rodents and ferrets and *Orthohepevirus* D, which infects bats, whilst *Piscihepevirus* includes cutthroat trout virus (CTV) [6]. There are currently seven recognized *Orthohepevirus* A genotypes. Genotype (G) I and GII only infect humans, while GIII and GIV infect a range of animal hosts, as well as humans. GV and GVI infect boars, while GVII infects camelids and humans. *Orthohepevirus* B has four proposed genotypes, *Orthohepevirus* C includes two genotypes (HEV-C1 and HEV-C2), while *Orthohepeviru*s D has a single genotype [6]. Additionally there is an unclassified moose HEV, which was recently identified in Sweden [7].

### HEV classification

An initial analysis of the HEV GI non-structural polyprotein in 1992 [8] indicated that the polymerase and helicase sequences were closely related to a plant virus, beet necrotic yellowing vein virus (BNYVV). BNYVV is a member of the newly proposed family *Benyviridae* and is a causative agent of rhizomania in sugar beet. This virus was first reported in Italy in 1966 [9], and was subsequently isolated in Japan in 1973 [10], and found to be transmitted by an obligate intracellular parasite protist vector, *Polymyxa betae* [11]. HEV and BNYVV both have positive sense single-stranded RNA (ssRNA (+)) genomes, although that of BNYVV is segmented into 4-5 separate RNA fragments [12], compared to the monopartite genome of HEV. Comparative analysis of the HEV RNA-dependent RNA polymerase (RdRp), methyltransferase and helicase protein sequences with other ssRNA (+) viral sequences also revealed a genetic relationship with rubella virus (RuBV) [8, 9]. These findings led to the initial inclusion of HEV within the “alpha-like” supergroup of viruses, based on similarities in their RdRp, helicase and methyltransferase amino acid sequences and also the presence of a putative papain-like cysteine protease [8, 13].

**Fig. 1:**
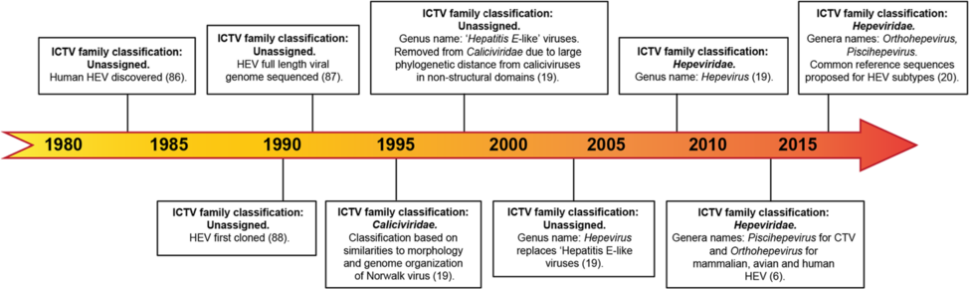
*Hepeviridae* classification timeline. The changing classification of *Hepeviridae* over time as determined by the International Committee on Taxonomy of Viruses (ICTV), with relevant works cited [6, 19, 20, 86–88]

A comparison of the genomic organization of HEV proteins with that of other viruses, and the absence of glycoproteins, led to an entirely new suggestion that HEV might have evolved from an ancestral calicivirus [8]. Surprisingly, this led to the inclusion of HEV within the *Caliciviridae* family, even though the *Caliciviridae* were already placed in the “Picorna-like” supergroup, and quite different to the “alpha-like” supergroup of viruses [8, 14, 15] (Fig. 1). Ultimately this led to some confusion over the ancestry of HEV, which was partially resolved in 1997, when the *Caliciviridae* were then subsequently organized into five genogroups, annexing HEV into a distinct sub-family by itself, but still within the *Caliciviridae* [16] (Fig. 1).

In 2000, phylogenetic analyses using more sequence data (including four different HEV strains), again analyzing capsid, helicase and RdRp conserved regions, moved HEV out of the *Caliciviridae* and into a separate, unassigned clade of indeterminate taxonomic position, but closer to the *Togaviridae* (which includes RuBV) [17], within the “alpha-like” supergroup. This finally resolved the problem of HEV being both a member of the “Picorna-like” supergroup and the “alpha-like” supergroup. As more and more sequences became available, HEV was seen to be more and more distinct from other viral genera and families, and it was accorded its own genus (*Hepevirus*) by 2004 and then its own family (*Hepeviridae*) in 2009 [18, 19]. In 2014, the taxonomy of the *Hepeviridae* was reviewed again and the family was divided into two new genera: *Orthohepevirus* and *Piscihepevirus*, as discussed earlier [6] and in 2016, Smith and colleagues proposed reference sequences for HEV subtypes within the *Orthohepevirus* genus [20]. Figure 1 illustrates the timeline of HEV classification through the International Committee on Taxonomy of Viruses (ICTV).

Despite scrutiny of the non-structural protein encoding region, the origin of the HEV capsid remained unresolved, since the *Benyviridae* are non-enveloped rod-shaped plant viruses [12], whereas HEV capsids are T = 3 icosahedral in shape, with an estimated 180 copies of the capsid protein [21, 22]. It was not until 2011, that surprisingly the HEV capsid protein was shown to be most structurally related to capsids from members of the *Astroviridae* family of vertebrate-infecting viruses [23]. Astroviruses, like HEV, also have a T = 3 icosahedral capsid, but which belong with the “Picorna-like” supergroup of viruses. To this day confusion over the ancestry of HEV is still apparent, as the non-structural protein-encoding region is classified within the “alpha-like” supergroup, in contrast to the structural region. Therefore, this study aimed to shed further insight into the evolution and origins of the *Hepeviridae* family in its entirety. We initially undertook both a phylogenetic approach and an updated analysis of the identity between the individual HEV proteins and other viral proteins. Here we provide evidence that the whole *Hepeviridae* family likely arose through an ancient recombination event, which could have occurred between plant and insect viruses, with the breakpoint at the junction that separates the non-structural from the structural encoding regions of the genome.

## Results

### Non-structural proteins

#### *Hepeviridae* evolution - Homology searches using the *Orthohepevirus* A HEV GI ORF1 polyprotein

**Fig. 2:**
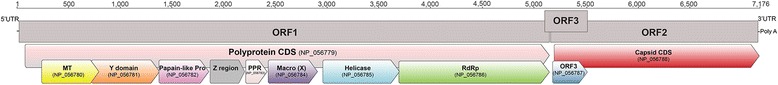
HEV GI protein organization of the encoded genome. The complete HEV GI prototype genome (GenBank accession number NC_001434), showing the individual ORFs 1 to 3 and their encoded proteins. The numbers along the top indicate nucleotide positions. Protein domains within the ORF1 polyprotein are shown along the bottom. The ORF1 region encoded between nucleotides 1780 to 2136 inclusive (termed region Z) has not been ascribed to any specific protein. The 3’ and 5’ untranslated regions and the poly A tail are indicated

**Fig. 3:**
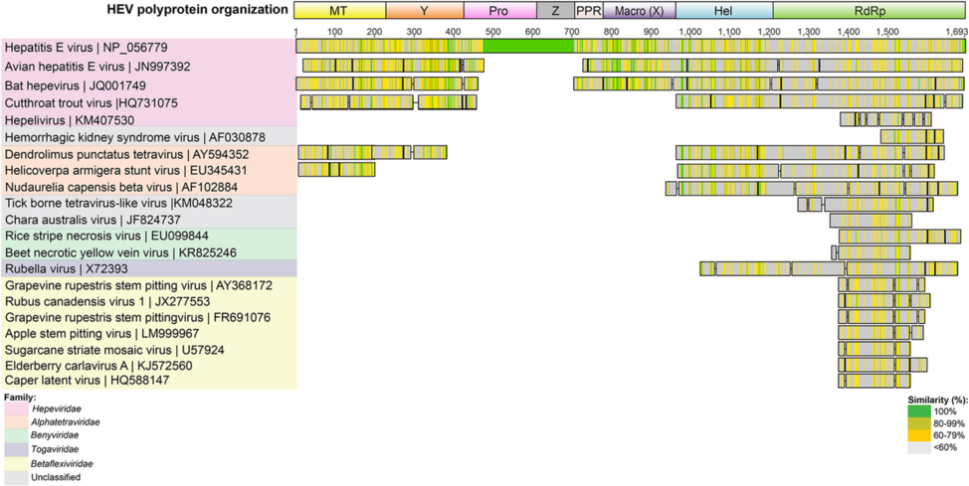
HEV GI ORF1 viral homologues. Selected *Hepeviridae* ORF1 protein sequences, and non-*Hepeviridae* protein sequences, were aligned to the translated prototype HEV GI ORF1 using Blastp. Homologues are organized into color-coded viral families. The organization of the individual proteins encoded by the HEV ORF1 is illustrated at the top. MT = Methyltransferase; Y = Y-domain; Pro = Papain-like cysteine protease; Z = Z region, PPR = Polyproline region; X = “X” or macro domain; Hel = Helicase; RdRp = RNA-dependent RNA polymerase. Green shading indicates 100 % similarity, light green when 80-99 %, yellow when 60-79 % and grey when <60 % similarity. Similarity is scored by all pairs of sites in any given column having a score according to the Blosum62 matrix, which exceeds a predetermined threshold value of ≥ 1. Positive scores occur between amino acids of similar physicochemical properties

To reveal close protein relatives within the *Hepeviridae* family, similarity searching of the GenBank database using the complete *Orthohepevirus* A HEV GI ORF1 polyprotein sequence (Figs. 2 and 3) (accession number NP056779) was initially undertaken. The closest non-human *Hepeviridae* ORF1-encoded homologues to the human HEV GI ORF1-encoded polyprotein, were the ferret *Orthohepevirus* C polyprotein (BAO57189; 57 % average amino acid identity over 1437 amino acids, in two fragments), followed by an avian *Orthohepevirus* B polyprotein (YP009001465; 49 % average amino acid identity over 1442 amino acids, in two fragments), bat *Orthohepevirus* D polyprotein (YP006576507; 49 % average amino acid identity over 1442 amino acids, in two fragments) and *Piscihepevirus* CTV polyprotein (YP004464917; 29 % average amino acid identity over 1434 amino acids, in two fragments) (Fig. 3, Table 1). Surprisingly, the searches using the HEV GI ORF1 polyprotein also highlighted a unique 275 amino acid region of the HEV genome (between amino acids 498-773), comprising the putative papain-like protease sequence (NP056782), the polyproline region (NP056783) and 119 residues of intervening sequence (here termed the Z region) (Fig. 2). This unique 275 amino acid region within ORF1 appeared to have little or no homology to any non-*Orthohepevirus* A sequence (Fig. 3 and Additional file 1: Figure S1).

**Table 1:** Individual HEV GI protein viral homologues

Protein	^a^Closest *Hepeviridae* homologue	E-value	Percent identity	1st-ranked Non-HEV viral homologue	Family/Genus	E- value	Percent identity	2nd-ranked Non-HEV viral homologue	Family/Genus	E- value	Percent identity
Polyprotein (ORF1) (*NP056779*)	*Orthohepevirus* C	0	57 %	^b^Helicoverpa armigera stunt virus (HASV)	*Alphatetraviridae* Omegatetravirus	1.74 × 10^-26^2.03 × 10^-7^	26 % 29 %	^b^Dendrolimus punctatus tetravirus	*Alphatetraviridae*/Omegatetravirus	1.28 × 10^-21^ 7.42 × 10^-8^ 7.42 × 10^-8^	25 % 25 % 20 %
Capsid (ORF2) (*NP056788*)	*Orthohepevirus* C	0	57 %	Porcine astrovirus	*Astroviridae*/Astrovirus	2.02 × 10^-12^	21 %	Chicken astrovirus	*Astroviridae*/Astrovirus	2.74 × 10^-12^	26 %
Phosphoprotein (ORF3) (*NP056787*)	None found	-	-	None found	-	-	-	None found	-	-	-
Methyltransferase (*NP056780*)	*Orthohepevirus* C	5.47 × 10^-65^	59 %	HASV	*Alphatetraviridae* Omegatetravirus	5.88 × 10^-7^	33 %	Frangipani mosaic virus	*Virgaviridae*/ Tobamovirus	6.50 × 10^-6^	30 %
Y domain (*NP056781*)	*Orthohepevirus* C	1.0 × 10^-96^	65 %	Hop latent virus	*Betaflexiviridae* Carlavirus	2.0 × 10^-18^	12 %	Blueberry scorch virus	*Betaflexiviridae* Carlavirus	6.0 × 10^-18^	12 %
PCP (Protease) (*NP056782*)	*Orthohepevirus* C	1.0 × 10^-10^	37 %	None found	-	-	-	None found	-	-	-
PPR (*NP056783*)	None found	-	-	None found	-	-	-	None found	-	-	-
Macro domain (X) (*NP056784*)	*Orthohepevirus* C	1.73 × 10^-45^	56 %	Aura virus	*Togaviridae*/ Alphavirus	4.61 × 10^-8^	36 %	Whataroa virus	*Togaviridae*/Alphavirus	5.95 × 10^-8^	36 %
Helicase (*NP056785*)	*Orthohepevirus* C	7.00 × 10^-87^	62 %	Turnip vein clearing virus	*Virgaviridae*/ Tobamovirus	6.04 × 10^-13^	31 %	Maracuja mosaic virus	*Virgaviridae*/ Tobamovirus	1.07 × 10^-12^	30 %
RdRp (*NP056786*)	*Orthohepevirus* C	3.64 × 10^-177^	59 %	Tick-borne tetravirus-like virus	Unclassified virus	7.82 × 10^-20^	33 %	Chara australis virus	Unclassified virus	2.98 × 10^-11^	34 %

^a^ Closest *Hepeviridae* homologue, excluding HEV taxonomic id (GenBank taxid #12161) sequences

^b^HASV has two distinct homologous fragments; *Dendrolimus punctatus* tetravirus has three distinct homologous fragments to HEV GI polyprotein

The closest non-*Hepeviridae* homologues to the *Orthohepevirus* A HEV GI ORF1 polyprotein were found to be from lepidopteran-infecting *Alphatetraviridae* family members (Fig. 3). Of the *Alphatetraviridae*, the highest scoring ORF1-encoded homologue by tBlastn or Blastp searching was *Helicoverpa armigera* (cotton bollworm) stunt virus (HASV) (genus *Omegatetravirus*), with homology to the MTase and helicase/RdRp regions (EU345431: 26 % and 29 % identity from two fragments comprising 823 amino acids; E-values = 1.74 × 10^-26^ and 2.03 × 10^-07^) (Fig. 3 and Table 1). Other *Alphatetraviridae* viruses with homology to the HEV ORF1 included *Dendrolimus punctatus* tetravirus (AY594352: 25 %, 25 % and 20 % identity from three fragments comprising 1,116 amino acids, E-values = 1.28 × 10^-21^ and 7.42 × 10^-8^), and *Nudaurelia capensis* beta virus (NP048059: 25 % and 20 % identity from two fragments comprising 1,104 amino acids) (Fig. 3).

Other noteworthy homologies were found, in the RdRp region, with a *Sclerotinia sclerotiorum* RNA virus [24], an unassigned dsRNA mycovirus infecting the plant pathogenic fungus *S. sclerotiorum*, (EU779934: 28 % average identity over 218 amino acids; E-value = 1.17 × 10^-9^); an unclassified tick-borne tetravirus-like virus [25] isolated from the ixodid hard tick *Dermacentor variabilis* (KM048322: 25 % average identity over 348 amino acids; E-value = 3.03 × 10^-17^) and the unclassified *Chara australis* virus [26], an ssRNA(+) virus infecting the charophyte algae *C. australis* var. *nobilis* (JF824737: 30 % average identity over 212 amino acids of the RdRp region; E-value = 4.73 ×10^-12^) (Fig. 3). The previously noted similarities between the HEV RdRp region and *Benyviridae* and *Togaviridae* viruses [8] were also observed in this study (Fig. 3).

Away from the *Alphatetraviridae* family, numerous homologues (1.0 ×10^-9^ > E-values > 1.0 ×10^-12^) were found within the *Betaflexiviridae* family of plant viruses (*Foveavirus*, *Carlavirus*) and *Virgaviridae* family of plant viruses (*Tobamovirus*), primarily with the helicase and RdRp regions of human GI HEV (Figs. 3, 5 and 6).

### *Hepeviridae* evolution – Homology searches using the *Orthohepevirus* A HEV GI individual protein sequences

**Fig. 4:**
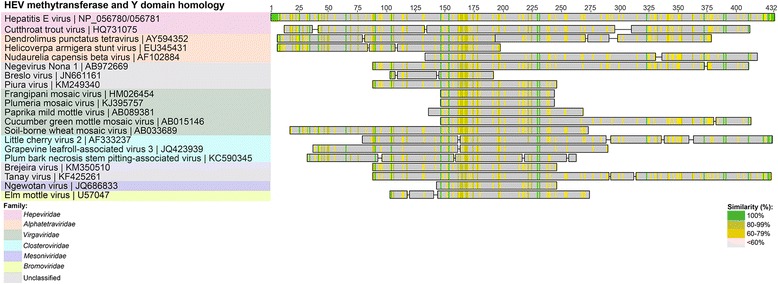
HEV GI Methyltransferase and Y domain viral homologues. Selected homologous sequences to concatenated HEV methyltransferase and Y-domain proteins identified using tBlastn search. Homologues are organized into color-coded viral families, as indicated in the legend. Shading as in Fig. 3

**Fig. 5:**
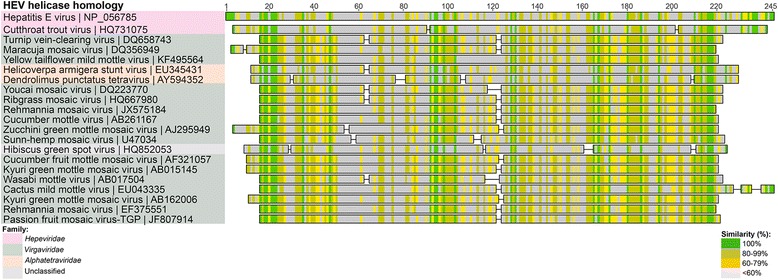
HEV GI Helicase viral homologues. Selected homologous sequences to concatenated HEV methyltransferase and Y-domain proteins identified using tBlastn search. Homologues are organized into color-coded viral families, as indicated in the legend. Shading as in Fig. 3

**Fig. 6:**
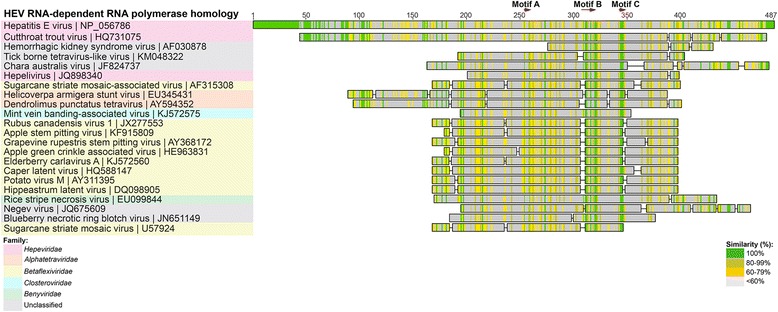
HEV GI RdRp viral homologues. Selected homologous sequences to HEV RdRp identified using tBlastn search. Homologues are organized into color-coded viral families. Shading as in Fig. 3

**Fig. 7:**
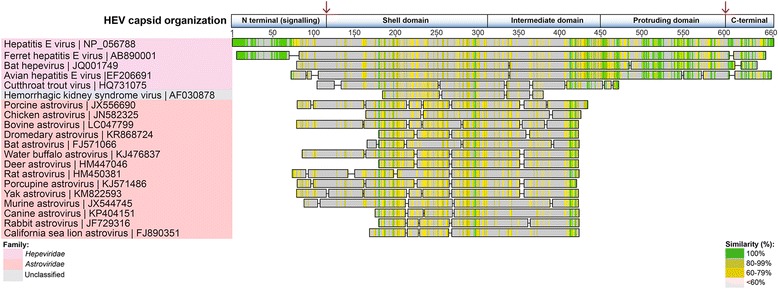
HEV GI capsid (ORF2) viral homologues. Selected homologous viral sequences to the HEV capsid protein, identified using tBlastn search. Homologues are organized into color-coded viral families. Shading as in Fig. 3. The HEV main capsid structural regions are illustrated above, to show regions of the HEV capsid with poor or no homologous sequence. S = shell domain; M = intermediate domain; *P* = protruding domain. The *N* (arginine/proline rich signal sequence) and C-terminal 52 amino acids of the protein sequence are absent in the mature capsid, as indicated by the *red arrows* [after Dryden et al. [60]]

The genomic organization of the HEV ORFs 1, 2 and 3, as illustrated in Fig. 2, comprises the following nine proteins in order from left to right, starting from the N-terminal: methyltransferase, Y-domain, papain-like cysteine protease, polyproline region, macro domain, helicase and RNA-dependent RNA polymerase (RdRp) from the polyprotein of ORF1, the capsid protein (ORF2) and phosphoprotein (ORF3). Additional tBlastn searches were undertaken using the individual *Orthohepevirus* A HEV GI protein sequences (accession numbers NP_056780 to NP_056788 inclusive) to identify any other, more distantly related viral protein homologues encoded by these regions. In particular, we wanted to determine whether the 275 amino acid region of the HEV GI genome between amino acids 498-773, described above, comprising the papain-like protease sequence, the polyproline region and Z region (the intervening sequence), had any non-*Hepeviridae* viral homologues, since it was poorly conserved within the *Hepeviridae* (Fig. 3 and Additional file 1: Figure S1). This search revealed a number of previously unidentified homologies to proteins from several viral families other than those previously recorded (such as the *Alphatetraviridae, Betaflexiviridae, Closteroviridae, Mesoniviridae* and *Virgaviridae* families; Figs. 3, 4, 5, 6 and 7) [8]. The individual protein homology searches are described below and the best hits are summarized in Table 1.

### Methyltransferase

Out of the *Hepeviridae*, the closest homologous sequence to the *Orthohepevirus* A HEV GI methyltransferase protein (MTase; accession number NP056780) was the *Orthohepevirus* C ferret HEV methyltransferase region (accession number BAO57185; E-value = 5.47 ×10^-65^, with an overall amino acid identity of 59 % over 182 amino acids) (Table 1). The closest non-*Hepeviridae* homologue from a tBLASTn search was found to be a methyltransferase protein from the aforementioned HASV (E-value = 5.88×10^-7^) (Fig. 4, Table 1); a 150 amino acid region with 33 % identity. Other members of the family such as *Nudaurelia capensis* beta virus (NBetaV, AF102884) were also observed to have weaker homology to the HEV MTase (Fig. 4). Representatives of the *Tobamovirus* genera (plant virus family *Virgaviridae*) were also weakly homologous to the HEV MTase (10^-6^ < E-value <10^-4^).

### Y-domain

The HEV Y domain (accession number NP056781), immediately upstream of the MTase region (Fig. 2) was first described by Koonin et al. [8], but has no described function. It was found to be well conserved within the *Hepeviridae*, and the nearest homologue was the corresponding ferret HEV Y domain of the *Orthohepevirus* C ferret HEV ORF1 polyprotein (BAO57189; E-value = 1.0 × 10^-96^, 65 % identity over the whole 217 amino acids (Table 1). Outside of the *Hepeviridae*, no significant identity was observed with the Y domain or any other protein (data not shown) using Blastp or tBlastn searches. However, a Delta-Blast search using a Blosum45 substitution matrix identified a region within the methyltransferase domain of Hop latent virus (NP066258; genus *Carlavirus*, of the *Betaflexiviridae* family of plant viruses), as the closest match, with an E-value = 2.0 ×10^-18^, with 12 % identity over 138 amino acids.

An InterProScan analysis of a concatenated methyltransferase and Y domain sequence showed that when combined, they contain a single region of 348 amino acids, corresponding to the Pfam member “*Vmethyltransf*” (PF01660, E-value = 7.5 ×10^-18^), suggestive of a larger contiguous MTase sequence, than that which is represented by NP_056780 alone. Pfam is a protein family database, comprising over 13,000 curated protein families that share significant homology as detected by the profile hidden Markov model method in the HMMER3 suite of programs [27].

An additional tBlastn search of the GenBank non-redundant viral database was undertaken, this time using the MTase and Y-domain concatenated sequence (Fig. 4). This identified the same hits as for the individual MTase and Y-domain searches above. However, the concatenated sequence search resulted in similar E-values, but with extended regions of homology for some viruses, such as NBetaV (AF102884), which now had a region of 323 amino acids (20 % identity, E-value = 4.0 ×10^-5^), aligned with the concatenated sequences, compared to 146 amino acids for the MTase alone (24 % identity, E-value = 1.37 ×10^-5^) (Fig. 4). In addition, with the extended sequence, new homologues were found with negeviruses, a recently described group of insect viruses [28]. The most homologous negeviral sequence was that of the polyprotein of Negevirus Nona 1, with 23 % identity over 313 amino acids (AB972669, E-value = 1.95 ×10^-4^); the homologous region corresponds to the putative methyltransferase domain [28] (Fig. 4).

### Papain-like cysteine protease

The HEV papain-like cysteine protease domain, of 160 amino acids (PCP; accession number NP056782) is not very well conserved, even within the *Hepeviridae*, and the nearest non-human *Hepeviridae* homologue, rat *Orthohepevirus* C PCP, has homology only at the N-terminal region of the PCP, with 42 % identity, from residues 33 to 78. An alignment of this region with other *Hepeviridae* family members illustrates the heterogeneity of this small region within the *Hepeviridae*, with low sequence identity across the family in this region (Additional file 1: Figure S1). Excluding the *Hepeviridae* from the search and using tBlastn, Blastp or PSI-Blast did not identify any significant homology with any other proteins in the GenBank database.

### Z Region

The Z region comprises amino acids 593-711 inclusive, of the *Orthohepevirus* A HEV GI ORF1 polyprotein sequence (NP_056779). This region appears to be unique across the *Orthohepevirus* A, with amino acid sequence identity of >68 % over 119 residues for viruses within the species. However, region Z is highly variable in both sequence and length outside *Orthohepevirus* A. No significant similarity of the Z region was detected outside the *Hepeviridae* family.

### Polyproline region

The polyproline region (PPR) of HEV, or hypervariable region (HVR) as it is also known (accession number NP056783), is an intrinsically disordered region (IDR), whose relaxed structure allows it to bind to multiple ligands, and be involved in the regulation of transcription and translation [29]. As its name suggests, the PPR contains a high level of proline residues, and is extremely variable in its sequence, even between *Orthohepevirus* A genotypes [30]. Despite significant inter-genotypic variation, within the *Orthohepevirus* A there is enough intra-genotypic conservation in the PPR to allow the creation of genotype-specific phylogenetic trees (data not shown). Blastp, tBlastn and PSI-Blast searches did not identify any significant homologues with other non-human *Hepeviridae* viruses, nor any other sequence, viral or non-viral, outside of the *Hepeviridae* (data not shown).

### Macro (X) domain

The macro domain (accession number NP056784) is involved in ADP-ribose metabolism and post-translational modifications. As well as being found in the *Hepeviridae*, homologues exist in the *Coronaviridae* and alphaviruses, as well as in bacteria and eukaryotes [31]. Within the *Hepeviridae*, the closest homologue to the HEV GI was the rat *Orthohepevirus* C macro domain (E-value = 1.73×10^-45^; with 56 % identity over 143 amino acids) (Table 1). The closest non-*Hepeviridae* homology was also seen between HEV and alphaviruses, such as Aura virus and Eastern equine encephalitis virus (EEEV) (AF126284; E-value = 4.61×10^-8^, with 36 % identity over 111 amino acids) (Table 1). Interestingly, the HEV macro domain also demonstrated similar identity with bacterial macro domains, such as that from *Clostridium spiroforme* (E-value = 1.62×10^-7^, with 32 % identity over 116 amino acids). The nearest eukaryotic homologue was the macro domain from the salmon flagellate protist pathogen *Spironucleus salmonicida* (EST47993; E-value = 5.0×10^-6^, with 32 % identity over 108 amino acids).

### Helicase

The HEV GI helicase protein (accession number NP056785) is also highly conserved within the *Hepeviridae* and has homology to non-*Hepeviridae* and non-viral helicases as well. The strongest non-human *Hepeviridae* Blast hit was found to be the helicase region of *Orthohepevirus* C ferret HEV ORF1 polyprotein (E-value = 7.0×10^-87^, with 62 % identity over 242 amino acids) (Table 1). The non-*Hepeviridae* viral helicase with the most identity to the HEV helicase was found to be that from the turnip vein clearing virus (Fig. 5), a *Tobamovirus* of the *Virgaviridae* family (E-value = 6.04×10^-13^, with 32 % identity over 250 amino acids). Another close homology was again observed in the helicase region from HASV (EU345431; E-value = 2.43×10^-12^, with 32 % identity over 250 amino acids) (Fig. 5).

### RNA-dependent RNA polymerase

The HEV GI RNA-dependent RNA polymerase (RdRp; accession number NP056786) is highly conserved within the *Hepeviridae*, with 84-89 % amino acid identity between the human HEV viruses, and 87-50 % within non-human *Hepeviridae* viruses. The strongest identity was found with the RdRp region of *Orthohepevirus* C ferret HEV polyprotein (JN998607; E-value = 3.64 ×10^-177^, with 59 % identity over 471 amino acids) (Table 1). The weakest *Hepeviridae* homology was with bat *Orthohepevirus* D and CTV, with 47 % and 34 % amino acid identity, respectively. The HEV RdRp also demonstrated homology to viral RdRps outside of the *Hepeviridae* family. Of the non-*Hepeviridae* homologues, the RdRp regions of two unclassified viruses had the highest identity to the human HEV RdRp; tick-borne tetravirus-like virus (KM048322; E-value = 7.82×10^-20^, with 33 % identity over 224 amino acids), and the algal virus *Chara australis* virus (JF824737; 2.98×10^-11^, with 34 % over 335 amino acids) [25, 26] (Fig. 6, Table 1). Other viral families also had RdRps that were highly homologous to HEV RdRp, including *Betaflexiviridae* and *Alphatetraviridae* (Fig. 6).

Another unclassified virus, Hemorrhagic kidney syndrome virus (HKSV) [32], (accession number AF030878), actually had higher homology to HEV RdRp (Fig. 6), than the above-mentioned Tick-borne tetravirus-like virus and *Chara australis* virus (E-value =1.66×10^-23^, with 39 % identity over 155 amino acids). HKSV was first identified in salmon in 2000, and originally described as a “togavirus-like” virus [32]. This partial sequence has a recognizable RdRp domain with the characteristic RdRp nucleotide motifs B and catalytic motif C [33], albeit separated by a stop codon (Fig. 6).

### HEV ORF2 (Capsid) protein

The *Orthohepevirus* A HEV GI capsid protein (encoded by ORF2; accession number NP056788) is well conserved within the *Hepeviridae* family, with the highest scoring non-human *Hepeviridae* homologue being the *Orthohepevirus* C ferret HEV capsid (AB890001; E-value = 0, with 57 % identity over 608 amino acids) (Fig. 7, Table 1). The next closest *Orthohepevirus* A homologue was that of bat *Orthohepevirus* D, (JQ001749; E-value = 3.94×10^-166^, with 55 % identity over 526 amino acids), followed by avian *Orthohepevirus* B (E-value = 6.55×10^-144^, with 49 % identity over 536 amino acids) (Fig. 7).

Interestingly, outside of the *Hepeviridae*, the chicken astrovirus capsid (JN582319; *Avastrovirus*, family *Astroviridae*) was found to be the most homologous sequence to the HEV GI capsid (E-value = 1.0 × 10^-6^, with 25 % identity over 278 amino acids) using a tBlastn search (Blosum62 matrix, word size 2, 3, or 6). Switching to the Blosum45 matrix, for aligning more divergent sequences, resulted in porcine astrovirus capsid being the most homologous sequence identified (JX556690; E-value = 2.02 × 10^-12^, with 21 % identity over 355 amino acids) (Fig. 7, Table 1). Other astrovirus capsid sequences were also found to be similarly homologous (Fig. 7), with E-values ranging from 10^-5^ to 10^-12^, and percentage amino acid identity ranging from 20-26 % (data not shown). In addition, the unclassified virus discussed above, HKSV, had a similar level of homology with the HEV capsid to those of the astroviruses (E-value = 9.95×10^-5^, 26 % identity over 164 amino acids). Additional PSI-Blast and Delta-Blast searches did not reveal any more significant homology to other capsid sequences from any other viral family or genera. A reciprocal tBlastn and PSI-Blast search using the translated chicken astrovirus capsid sequence JN582319, identified HEV capsid protein as its closest homologue (27 % identity over 278 amino acids; E-value = 3.0 × 10^-6^).

### HEV ORF3 phosphoprotein

The function of the small phosphoprotein encoded by HEV ORF3 (accession number NP056877) is not fully understood, but it plays a role in virion release from infected cells, and has also recently been found to inhibit nuclear factor-κB signaling [34]. It is conserved within the human-infecting *Orthohepevirus* A genotypes, but not within the *Hepeviridae* family, even though viruses from the *Orthohepevirus* B, C and D, and CTV all have small, unrelated ORFs (presumed phosphoproteins) in the same part of their respective genomes. Delta-Blast and PSI-Blast searches did not reveal any other homologous proteins (viral or non-viral).

### *Hepeviridae* evolution – capsid and RdRp phylogeny indicates ancient recombination events

**Fig. 8:**
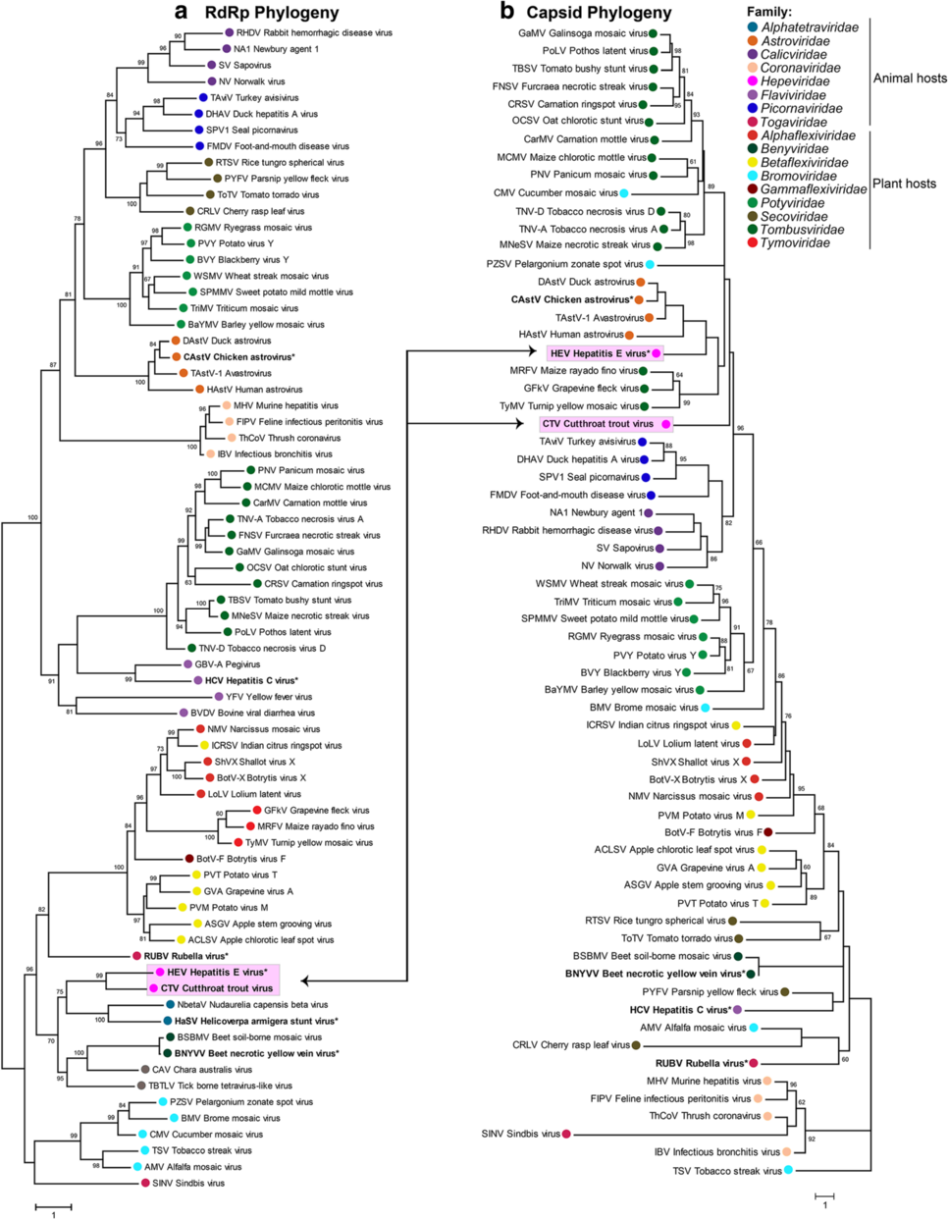
Rooted ssRNA (+) viruses capsid and RdRp phylogeny. **a** Midpoint rooted phylogenetic tree of 72 ssRNA (+) RdRp sequences, aligned by MAFFT and phylogeny performed using PhyML. Branch labels are bootstrap values. **b** Midpoint rooted phylogenetic tree of 65 ssRNA (+) capsid sequences, aligned by MAFFT and phylogeny performed using PhyML. Branch labels are bootstrap values. Recombination events within the *Hepeviridae* are shown by black arrows. Scale bars indicate number of nucleotide substitutions per site

**Fig. 9:**
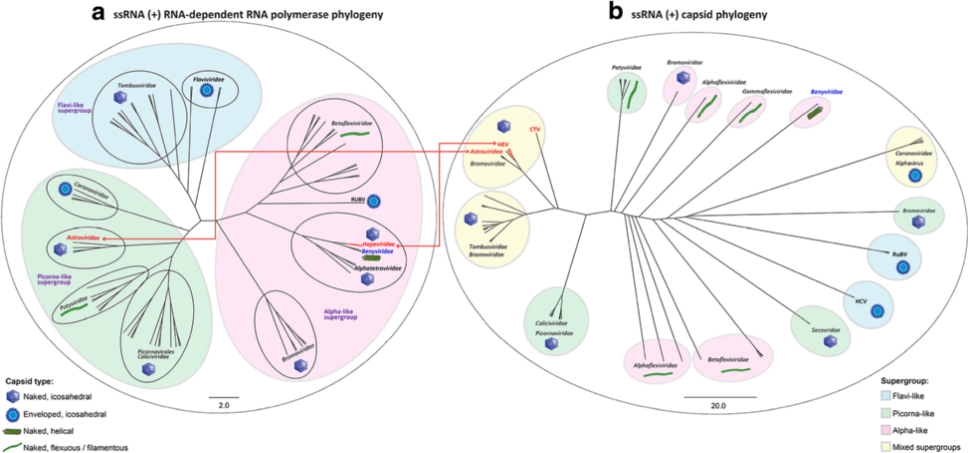
Unrooted ssRNA (+) viruses Capsid and RdRp phylogeny. Unrooted phylogenetic tree of ssRNA (+) (**a**) RdRps and (**b**) capsids, aligned by MAFFT and phylogeny performed using PhyML. Shading indicates the viral supergroup to which clusters belong, as indicated by the key. *Red arrows* demonstrate the disparate genetic relationships for non-structural and structural proteins between supergroups, for viruses belonging *Hepeviridae* and *Astroviridae*. The capsid type of each cluster or group is depicted by an icon as indicated in the legend. Scale bars indicate number of nucleotide substitutions per site

An obvious disparity was revealed by the above analyses; the *Orthohepevirus* A HEV GI RdRp encoded from ORF1 is clearly related to viruses from families within the “alpha-like” supergroup, whilst the capsid encoded by ORF2 appears to be related to viruses of the *Astroviridae* family that falls within the “Picorna-like” supergroup of viruses. In order to understand these disparate homologies, a phylogenetic analysis of the HEV RdRp and capsid protein sequences was undertaken in relation to other viruses. RdRp and capsid sequences (Additional file 2: Table S2) from most of the type strains of class IV (+) ssRNA viruses were aligned by MAFFT, and phylogenetic relationships were inferred using PhyML to generate rooted and unrooted trees (Figs. 8 and 9).

The RdRp tree places the HEV RdRp and CTV RdRp together, forming a small cluster of RdRps with those from the *Alphatetraviridae* and *Benyviridae* families. The unclassified tick-borne tetravirus-like virus and *Chara australis* virus also groups with this cluster (Fig. 8). This small cluster’s next nearest neighbor is RuBV. All of these group together, forming one of the main clusters within the “alpha-like” supergroup of viruses, which can be clearly seen in the unrooted tree (Fig. 9).

In contrast to the RdRp phylogeny, the rooted capsid tree places the HEV capsid within a cluster with the *Astroviridae* family of viruses (Fig. 8). The *Astroviridae* capsid is also closely related to the *Tymoviridae* family of monopartite, T = 3 icosahedral plant viruses. Although the HEV RdRp sequence groups with members of the *Benyviridae* and RuBV, there is no relationship between the *Hepeviridae* capsids and capsid sequences from these viruses, which group well away from the *Astroviridae* cluster (Fig. 8). Indeed, capsids from viruses of the *Benyviridae* are helical in architecture, not icosahedral. This relationship can also be seen more clearly in the unrooted capsid tree (Fig. 9). In order to reduce the potential effects of saturation [35] due to the large scale phylogeny of all (+) ssRNA capsids (and their rapid mutation rates), an extended set of capsid sequences was analyzed again, this time including more *Hepeviridae* and *Astroviridae* sequences, but with fewer, more closely related families (Additional file 3: Figure S2). This analysis included 223 representatives from the *Astroviridae*, *Benyviridae*, *Bromoviridae*, *Caliciviridae*, *Hepeviridae*, *Tombusviridae* and *Tymoviridae* families (Additional file 2: Table S3). All of the families form individual clusters, except for the *Bromoviridae* strains. There is good branch support for several of the intrafamily clusters (*Hepeviridae*, *Tymoviridae*, *Benyviridae* and *Caliciviridae*), but this is weaker (<60 %) for the other intra- and interfamily clusters (Additional file 3: Figure S2). However, the *Astroviridae* and *Hepeviridae* group together, with the *Tymoviridae* linked to the *Hepeviridae* after apparent divergence from the *Astroviridae* cluster (Additional file 3: Figure S2).

## Discussion

### Homology searches using the HEV GI proteins

The first part of this study has highlighted four key areas that lead to a greater understanding of the origins and evolution of the *Hepeviridae* as a whole, with a focus on the prototype virus HEV GI in particular, and these are discussed in more detail below.

### MTase, Y-domain, helicase and RdRp

The well-characterized domains with recognizable homology to other viral proteins include the MTase, Y-domain, helicase and RdRp domains. Of these, the Y-domain was originally identified as a distinct domain with weak homology to genomic regions in RuBV and BNYVV [8]. However, no function was ever ascribed to it. Since the preceding methyltransferase domain (NP056780) shares the same Pfam domain classification as the Y-domain, it seems more than likely that the Y-domain is actually just a C-terminal domain of a larger methyltransferase protein. Therefore, the MTase (NP056780) and Y-domain (NP056781) sequences should be considered together as one methyltransferase protein.

Recently, a secondary structure comparison of 50 viral genera identified an “iceberg” region, downstream of the “core” region within viral methyltransferase-guanylyltransferase (MTase-GTase) capping enzymes, to which the HEV Y-domain was also included [36]. Experimental evidence for the iceberg region or Y-domain’s functional role has been demonstrated using C-terminal deletions of the Sindbis virus MTase-GTase [37]. Here, even relatively small C-terminal deletions abolished MTase-GTase activity, demonstrating that the core region alone is insufficient for all capping enzyme activities. This is further evidence that the MTase domain, together with the Y domain, forms either a single protein or single domain.

The individual searches with the *Orthohepevirus* A HEV ORF1 polyprotein and the individual MTase, Y-domain, helicase and RdRp proteins suggests that the majority of the ORF1 polyprotein of viruses from the *Hepeviridae* (comprising the MTase-Y-domain, helicase and RdRp domains) share a common ancestor with the *Alphatetraviridae* family, and that ancestor in turn shares a common ancestor to the *Virgaviridae*/*Benyviridae*/*Betaflexiviridae* plant viral families. Other features of the HEV genome were probably acquired by horizontal gene transfer (polyproline-macro domain) or evolved later as a result of host-virus interactions (polyproline region).

### Papain-like cysteine protease, polyproline region, Z region and macro domain

**Fig. 10:**
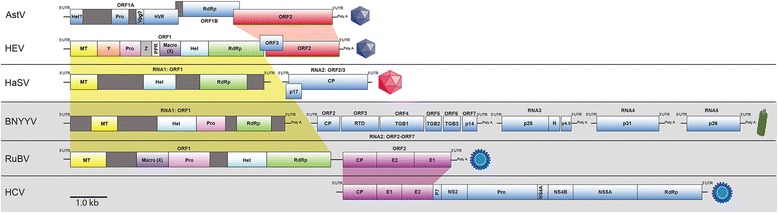
HEV genome comparisons of HEV, as a representative virus of the *Hepeviridae*. Comparison of the HEV genome architecture with other viral genomes, where colored shading indicates the relationship between different non-structural and structural encoding regions. Regions encoding homologous proteins are color-coded demonstrating the organization of the various domains in the genomes. Genomes shaded in grey are more distally related. Capsid types are represented by an icon, purple is T = 3 icosahedral, pink is T = 4 icosahedral, green is naked helical and blue represents enveloped icosahedral virions. A scale bar in Kb is shown

The putative papain-like cysteine protease domain, polyproline region, Z region and macro domain are the poorly characterized domains with little or no homology to other viral proteins. In the HEV genome these are grouped together, bisecting the four recognized proteins (Figs. 2 and 10). In addition, we recognized an uncharacterized region of 119 amino acids upstream of the polyproline region, which we have termed the Z region (Figs. 2 and 10), as there are already domains termed Macro (X) and Y present within the HEV genome.

The existence of the HEV protease was first predicted by Koonin et al. [8], based upon alignments with RuBV and BNYVV viruses. However, the alignment is poor, and subsequent advances in sequence alignment and enlarged databases have not led to the identification of any significant homologues to the HEV protease domain. Indeed, one of the most contentious issues regarding HEV is whether the ORF1 polyprotein is processed at all, either by its own protease or by a host protease. Evidence for in vitro processing of the HEV ORF1 has been reported in *E. coli,* in human HepG2 and S10-3 cells [38–40], in baculovirus cells [41] and using vaccinia virus expression systems [42]. However, in all of these studies, the levels of the putative processed proteins are low, and in some cases could be explained by host-cell protease degradation. A recombinant HEV protease purified from *E. coli* was found to be active on both the HEV capsid protein and the HEV polyprotein, although an inhibitor screen of this protease’s activity led to the conclusion that it was a chymotrypsin-like protease, not a papain-like cysteine protease [43]. However, other studies looking at ORF1 processing in human and hepatic cells reported little or no processing [44, 45]. This data is consistent with the fact that, a specific histidine residue (H590), a key part of the catalytic dyad in papain-like cysteine proteases, is absent in the HEV enzyme [46]. Pertilla et al. [45] also suggests that the HEV polyprotein is not processed, and functions in a similar manner to the large multifunctional polyproteins of some plant viruses within the “alpha-like” supergroup, consistent with its phylogenetic clustering (Figs. 8 and 9) [47]. Conversely, Parvez and colleagues used *in silico* modelling of RuBV and HEV proteases to identify a different catalytic dyad in the putative HEV papain-like protease. This study proposed that the catalytic dyad is C434-H443, which is homologous to the C1152-H1273 catalytic dyad in the RuBV protease [48]. Using mutational analyses, the conserved H443 was shown to be is essential for HEV viability [48]. In the current study, the H443 residue was found to be conserved within the *Orthohepevirus* A, but not across the *Hepeviridae* family. Irrespective of the functionality (or not) of the protease, its origin remains obscure due to the lack of any significant identity to non-*Hepeviridae* sequences.

From this study, it is also interesting to note that this “protease” region, which lies between the MTase-GTase and the polyproline region, is approximately the same size across members of the *Hepeviridae*. Yet the amino acid identity is so low that no genetic relationship is apparent (Additional file 1: Figure S1). This suggests a common functional enzyme does not exist. Instead, perhaps this is a region involved in host-specific interactions. Whether this domain is a degenerate partially functional or non-functional protease domain, or an entirely different domain or protein, remains to be clarified.

One other study has reported deubiquitination activity, a key target for viral infection [49], associated with a recombinant protein comprising the methyltransferase, Y-domain and protease domain (amino acids 22-592) of HEV ORF1 [50]. A later study showed that a recombinant HEV protease domain, when expressed in HEK293T cells, resulted in reduced IFNβ mRNA production and deubiquitination of RIG-I and TBK-1 [51]. Since cysteine proteases are significantly represented as a subfamily within the larger deubiquitinase (DUB) enzyme family [52], this could well be the main function, even perhaps the only function, for the protease domain.

The macro domain of HEV is homologous to those found in alphaviruses and bacteria, with slightly less similarity to eukaryotic and archeal macro domains, but the differences in homology are not significant enough to identify unambiguously the origin or common ancestor of this region. It is also unclear whether the HEV macro and protease domains together derive from the same ancestral region, as is the case for the *Coronaviridae* and alphaviruses [14], where the macro domain is associated with the protease domain. Macro domains in animal cells have been associated with ADP-ribose metabolism, and post translational protein modification, but few viral families possess macro domains, and in those viral families that do, the macro domains do not appear to be related to any specific feature shared by these viruses (*Coronaviridae*, alphaviruses, *Hepeviridae*) [31]. Poly(ADP)-ribosylation is a post-translational modification of proteins in cells, which can be triggered by viral infection, and acts as an apoptosis-inducing signal [53], and the HEV macro domain has been shown to bind poly(ADP), suggesting a potential role in preventing apoptosis in infected cells. Expression of a recombinant HEV macro domain in HEK293T cells inhibited poly(IC)-induced IRF3 phosphorylation, indicating that one of the HEV macro domain’s function is as an IFN antagonist [51].

The polyproline region (PPR) was originally described as a protein hinge [8], but more recent studies have shown it to be the only region within ORF1 under positive selection, and has multiple ligand-binding motifs [29]. The PPR is also involved in viral replication and adaptation, including host-specificity, but not infectivity [54, 55]. An increase in correlated genetic heterogeneity in the macro and polyproline regions has been observed in chronic HEV infection, relative to patients who cleared the virus, suggesting that these two domains are linked in some way in modulating the immune response [56]. Apart from the *Hepeviridae*, only rubiviruses have a similar arrangement of a PPR upstream of a macro domain, and the rubivirus PPR is also highly diverse genetically, and under positive selection [29]. It is conceivable that ancestral rubiviruses and *Hepeviridae* viruses at some point acquired or exchanged a PPR-macro domain, which has retained homology in the macro domain, for its shared function in host-virus interactions, but the PPR domain has evolved rapidly due to its host-specificity role.

The Z region of the *Orthohepevirus* A HEV GI ORF1 is unique across the species and as such, resembles an equivalent alphavirus unique domain (AUD); a region of the alphaviral non-structural protein coding region that has strong sequence identity within alphaviruses, but not outside the genus [57]. Like the AUD, the Z region is highly variable in both sequence and length outside *Orthohepevirus* A, but is conserved within the *Orthohepevirus* A group (>68 % identity across 119 residues). It is possible that the AUD-like region in *Orthohepevirus* A viruses has evolved with changes in host tropism, thus losing any conservation with other members of the *Hepeviridae* over time. Similar to the Z region in *Orthohepevirus* A, in alphaviruses a PPR follows the AUD, however unlike HEV both domains are downstream of the macro domain (Fig. 2). In the majority of *Orthohepevirus* A sequences, the Z region and PPR are both upstream of the macro domain and could have shifted through a translocation event. One exception within *Orthohepevirus* A is rabbit HEV, and outside *Orthohepevirus* A exceptions include ferret and rat HEV (*Orthohepevirus* C) and CTV (*Piscihepevirus*) which all have a small PPR region at the carboxyl end of the macro domain. This polyproline rich region was originally reported to be an insertion of an inert spacer sequence into the rabbit and rat genomes [58], but could in fact be a remnant from an incomplete PPR translocation event.

### Capsid

Whereas the majority of the HEV ORF1-encoded polyprotein and those from the *Hepeviridae* family in general clearly have plant/insect viral ancestry, the HEV capsid sequence appears to have none. Analysis of the capsid spike or projection domain of the human astrovirus serotype 8 (human astrovirus; Yuc8 strain, genus *Mamastrovirus*) using the DALI protein homology server [59] identified the HEV capsid P2 domain as the closest structural homologue [23].

Electron cryomicroscopy also showed that the human astrovirus infectious virions and immature virions were remarkably similar to that of HEV in terms of size, shape and architecture [22, 60]. It is interesting to note that, given the similarity in capsid structural size, the genomes of HEV (~7.2 kb) and astroviruses (6.8-7.1 kb) are also similarly matched in size. A subsequent study [61] found that the turkey astrovirus spike protein (turkey astrovirus-2; genus *Avastrovirus*) was a structurally closer match to the HEV capsid, than was found with the previous study involving the human astrovirus capsid spike [60]. The present study however, showed that the porcine astrovirus capsid protein sequence was the closest homologue to the HEV capsid sequence, closely followed by the chicken astrovirus capsid (Fig. 7), and all the homologous *Astroviridae* capsids sequences had E values between 10^-5^ – 10^-12^. Moreover, no other viral family capsid sequences had any significant homology to the HEV capsid. A reciprocal Blast search using a chicken astrovirus capsid sequence identified the HEV capsid as the closest homologue, and no other viral family capsid had any significant identity. Irrespective of which contemporary astrovirus sequence is most closely related to contemporary HEV capsids, it seems that the *Hepeviridae* and the *Astroviridae* viruses share a common ancestral capsid sequence.

Surprisingly, it was also found that the *Tymoviridae* and *Hepeviridae* capsid sequences have a common ancestor too, which diverged after the *Hepeviridae*-*Astroviridae* split (Additional file 3: Figure S2). However, the *Hepeviridae* and *Tymoviridae* capsid sequences do not have any significant homology, but are linked phylogenetically, which could be indicative of the (plant-infecting) *Tymoviridae* capsid sequences evolving faster under different evolutionary pressure(s), than the avian/mammalian-infecting *Hepeviridae* or *Astroviridae* capsids.

### ORF3 Phosphoprotein

The absence of any viral homologues to the phosphoprotein encoded by ORF3, even within the *Hepeviridae* would suggest that ORF3 evolved relatively recently from an overprinting mechanism [62], utilizing a previously unused ORF within ORF2. ORFs 2 and 3 are bicistronic, with the two start codons closely spaced in different reading frames [63]. ORF3 has evolved to be essential for virulence [64]. Viruses with this type of ORF3 likely occurred after mammalian *Orthohepevirus* A virus diverged from the common *Hepeviridae* ancestor and it is possible that ORF3 could have driven host tropism and host restriction [65]. Similar overprinted genes like ORF3, have been identified in numerous other viruses [66]. However, *Astroviridae* capsid sequences have not been shown to have overlapping proteins expressed from alternative ORFs, and it is seems highly improbable that ORF3 was independently acquired and integrated into the capsid sequence.

### Proposed evolutionary scenario for the *Hepeviridae*

There are few reports in the literature regarding the evolution of contemporary viruses from two or more disparate ancestors. Although there are few examples, one in particular is remarkable; the ssDNA viruses of the *Bidnaviridae* family (infecting the silkworm *Bombyx mori*) contain genes derived from *Parvoviridae* (ssDNA viruses), *Reoviridae* (dsRNA viruses), *Baculoviridae* (dsDNA viruses) and polintoviruses [67]. In addition, a recent metagenomics study of viral diversity in a lake has identified a unique hybrid virus, where an ssRNA viral capsid sequence has integrated into a circovirus-like DNA virus (RNA-DNA hybrid virus from Boiling Springs Lake, or “BSL-RDHV”) [68]. The capsid sequence in BSL-RDHV is most closely related to the capsids from multipartite nodaviruses, which mostly infect insects and fish, and to monopartite *Tombusviridae* capsids (plant viruses), and the remainder of the BSL-RDHV genome resembles circoviruses (infecting birds and mammals) [68]. RNA-RNA recombination in plant viruses and other viruses, and the relevant molecular mechanisms are well-known and well-documented [69]. Some families show high rates of recombination (*Picornaviridae*), and others low rates (*Flaviviridae*), or non-existent/undetectable (*Leviviridae*), nonetheless recombination is vital for generating population variability and maximizing survival [70].

There are few if any published reports of interfamily capsid exchange in the ssRNA (+) viruses, notwithstanding the fact that such an exchange would in all likelihood require similar sized genomes in order to package properly. The most parsimonious evolutionary scenario, with regard to the exchange, or acquisition of capsid sequences by an ancestral *Hepeviridae* virus, might be that an ancient ORF1 RNA sequence, from a plant/insect virus ancestral to the *Alphatetraviridae*/*Betaflexiviridae*/*Virgaviridae*/*Benyviridae* families, underwent recombination with an ancestral *Astroviridae* capsid RNA sequence, resulting in a recombinant virus that had (fortuitously) similarly sized genome of a plant/insect virus. This virus likely retained capsid sequence packing signals and was able to be subsequently packaged into an astrovirus-like icosahedral capsid. Such a recombination event resulted in the ancestral *Hepeviridae* hybrid virus, comprising an astroviral capsid from the Picorna-like super-group, and a plant/insect non-structural polyprotein from a virus within the very different alpha-like super-group (Fig. 10). An obvious prerequisite of the above events is that the ancestral viruses involved infected the same host simultaneously; whether this permissive host was a plant, insect or possibly even a vertebrate is at present unclear.

The ancestral *Hepeviridae* ORF1 probably comprised (from N- to C-terminal) a methyltransferase, “protease”, helicase and RdRp regions. At some time a macro domain was acquired, probably by horizontal transfer, although the putative source is unclear, given the similar, (albeit low) identity to bacterial and alphaviral homologues. Whether this acquisition occurred before, or after the capsid exchange is unclear. This acquisition may have been accompanied by the “protease” region, since these two domains are often closely associated, although no functional link between these domains has yet been demonstrated [14]. The PPR could also have been associated with the macro domain as discussed above. The orientation of the protease and macro domains in *Hepeviridae* viruses correspond to that seen in alphaviruses, but is the reverse of RuBV, whereas the PPR-macro domain organization in *Hepeviridae* matches that of rubiviruses, but not alphaviruses in general (which have a much smaller PPR region or lack the PPR altogether) [8, 47] (Fig. 10). Later still, the ORF3 phosphoprotein evolved from an alternate reading frame within the capsid sequence, which could have been present in the capsid region prior to recombination, or evolved afterwards. Subsequently, the evolution of ORF3 could have been influenced by host tropism, leading to the possible evolution of individual species within the *Hepeviridae*.

Intriguingly, duck astrovirus is unique among the *Astroviridae*, in causing viral hepatitis, indicating a tropism for hepatocytes, whereas all other astroviruses cause gastroenteritis [71]. It is interesting to speculate that the ancestral virus, from which viruses of the *Astroviridae* and *Hepeviridae* obtained their capsids, had a similar tropism. This could help to explain how the descendent capsid of an enteric virus is now used by a virus that causes human hepatitis.

It is also noteworthy that analysis of the *Astroviridae* capsid and RdRp sequences indicate the possible occurrence of a second ancient inter-viral family recombination event. The astrovirus RdRp sequences are unrelated to those from the *Hepeviridae* family, but are most closely related to those from viruses of the *Potyviridae*; a large family of non-enveloped plant-infecting viruses [72] with a flexible filamentous rod-shaped virion (Figs. 8 and 9). These capsids share no homology whatsoever with the icosahedral astrovirus capsid. This likely indicates that an ancestor of the *Potyviridae* acquired a distinct *Astroviridae*-like capsid (i.e., from a different viral family), indicating a recombination event at the non-structural/structural junction, that led to the genesis of the *Astroviridae*.

Koonin et al., [8] first proposed that HEV (and hence subsequently the whole family) had a genetic relationship in the non-structural encoding region of its genome with viruses from the *Togaviridae*, including RuBV and BNYYV; this is depicted in Fig. 10. In contrast to the *Hepeviridae*, the *Togaviridae* (including RuBV), have an alpha-like non-structural genomic region, but a flaviviral-like structural region of their genome (Fig. 10). Given that the two aforementioned recombination predictions likely led to the genesis of the *Hepeviridae* and the *Astroviridae*, it is tempting to speculate that the entire *Togaviridae* family could also have emerged this way. It may have occurred through a recombination event at the non-structural/structural border between viruses from families representing different viral super-groups; flavi-like and alpha-like super-groups.

## Conclusion

This study used protein identity searches using the prototype HEV virus ORF1 amino acid sequence to highlight four key areas that lead to a greater understanding of the origins of HEV, the possible functions of its encoded proteins and their relationship to other protein families. This study has also shed additional light on the intriguing evolution and ancestry of several viral families. We show that both the *Astroviridae* and *Hepeviridae*, and possibly the *Togaviridae*, have undergone ancient recombination events at the junction between the non-structural and structural encoding regions of the genome, resulting in their creation. It is likely other families have emerged this way and this could be uncovered with careful analysis of the ever-growing number of new viral sequences being accrued. Finally, based on the results found herein, we hypothesize that viruses from the alpha-like super-group, which usually infect plants and insects, gain an entirely new structure through recombination to enable tropism into new hosts within the animal world.

## Methods

### *Hepeviridae* genomic sequences

A total of 301 *Hepeviridae* genomes were identified and downloaded from GenBank (October, 2015). Duplicate sequences were removed using the ElimDupes tool on the HIV sequence database (https://hcv.lanl.gov/content/sequence/ELIMDUPES/elimdupes.html). *Orthohepevirus* B, C and D genomes, along with the *Piscihepevirus* CTV genome, were removed. Rabbit HEV (GIII), wild boar HEV (GV), and camel HEV (GVII) sequences were included, but wild boar HEV (GVI) and Moose HEV (unclassified) sequences were omitted. Incomplete sequences, patent sequences of cDNA, recombinant infectious clone constructs and sequences from *ex vivo* passaged isolates were also excluded, resulting in 228 non-redundant HEV genomes (Additional file 2: Table S1).

### Identity searching of *Orthohepevirus* A HEV GI proteins

To identify homologous *Hepeviridae* and non-*Hepeviridae* viral sequences, individual *Orthohepevirus* A HEV GI protein sequences were used in tBlastn and Blastp searches of the GenBank databases [73], using the built-in interface in the software package Geneious 9.0.3 [74]. Specifically, protein sequences derived from the prototype HEV Xinjiang strain (accession number NC_001434) [75] were used, corresponding to the methyltransferase (NP056780), Y-domain (NP056781), papain-like cysteine protease (NP056782), hypervariable region (HVR) or polyproline region (NP056783), X (macro) domain (NP056784), helicase (NP056785) and RNA-dependent RNA polymerase proteins (RdRp, NP056786), shown together in Fig. 2. In addition, searches were also conducted with the complete polyprotein of ORF1 (NP056779), the capsid protein from ORF2 (NP056788) and the phosphoprotein encoded by ORF3 (NP056787) [75]. An “expectation” or “expect value” (E-value) threshold of < 1× 10^-4^ was used to eliminate weak and random similarities between the query and target sequences [76].

### Homology of HEV GI protein sequences searching by PSI-Blast, Delta-Blast and InterProScan

The individual human HEV GI protein sequences and the HEV ORF1 polyprotein sequences described above (Fig. 2), were also used to screen the non-redundant GenBank protein database for distant homologues, using the position-specific iterated Blast (PSI-BLAST) server at http://blast.ncbi.nlm.nih.gov/ [77]. Initial setup parameters comprised the default settings, using either BLOSUM45 or BLOSUM62 as the initial substitution matrix, word size 2 or 3, up to 5 iterations, and masking of low complexity regions. In addition, searches were made using the same parameters on the Domain Enhanced Lookup Time Accelerated Blast, or Delta-Blast server. This server queries the NCBI conserved domain database (CDD) to construct a scoring matrix prior to searching the protein sequence database [78]. Individual HEV GI protein sequences were also analyzed for protein function against the InterPro protein families’ database, using the InterProScan plugin in Geneious [79, 80].

### Multiple sequence alignment and phylogenetic analysis

Capsid and RdRp protein sequences from 104 representative positive sense, single stranded RNA viruses (ssRNA (+)) were collated from GenBank (Additional file 2: Table S2). These sequences were aligned using MAFFT [81], using the embedded algorithms in Geneious. The best-fit models for amino acid substitution for the phylogenetic analysis were determined by analyzing the capsid and RdRp alignments using ProtTest 3.3 [82]. Phylip files of the alignments were submitted to the ATGC Server at http://www.atgc-montpellier.fr/ to build the phylogeny using PhyML [83], using the LG substitution model [84] for RdRp alignments, and the VT substitution model for capsid alignments [85], with SPR and NNI tree improvements and 100 bootstraps.

## Additional files

Additional file 1: Figure S1.
*Hepeviridae* protease alignment. Alignment of the 3’ terminal of the Y-domain, protease domain and polyproline region of HEV GI and the three closest *Hepeviridae* homologues, using MAFFT. (TIF 2501 kb)
Additional file 2: Table S1. List of *Hepeviridae* genomes analyzed. **Table S2.** List of capsid and RdRp sequences used for Figs. 8 and 9. **Table S3.** List of 223 genomic sequences from capsid sequences used for creating additional Fig. 2. (DOCX 122 kb)
Additional file 3: Figure S2. Extended capsid phylogeny. Midpoint rooted maximum likelihood phylogenetic tree of 223 capsid sequences (Additional file 2: Table S3) from seven families, aligned by MAFFT and phylogeny performed using PhyML. Red values: bootstrap scores > 60 %; Black values: substitutions per site. (TIF 914 kb)
