# Supplementary figures and images for: Ancient recombination events and the origins of hepatitis E virus
Source: BMC Evol Biol. 2016 Oct 12;16:210. doi: 10.1186/s12862-016-0785-y (PMC5062859; doi:10.1186/s12862-016-0785-y)

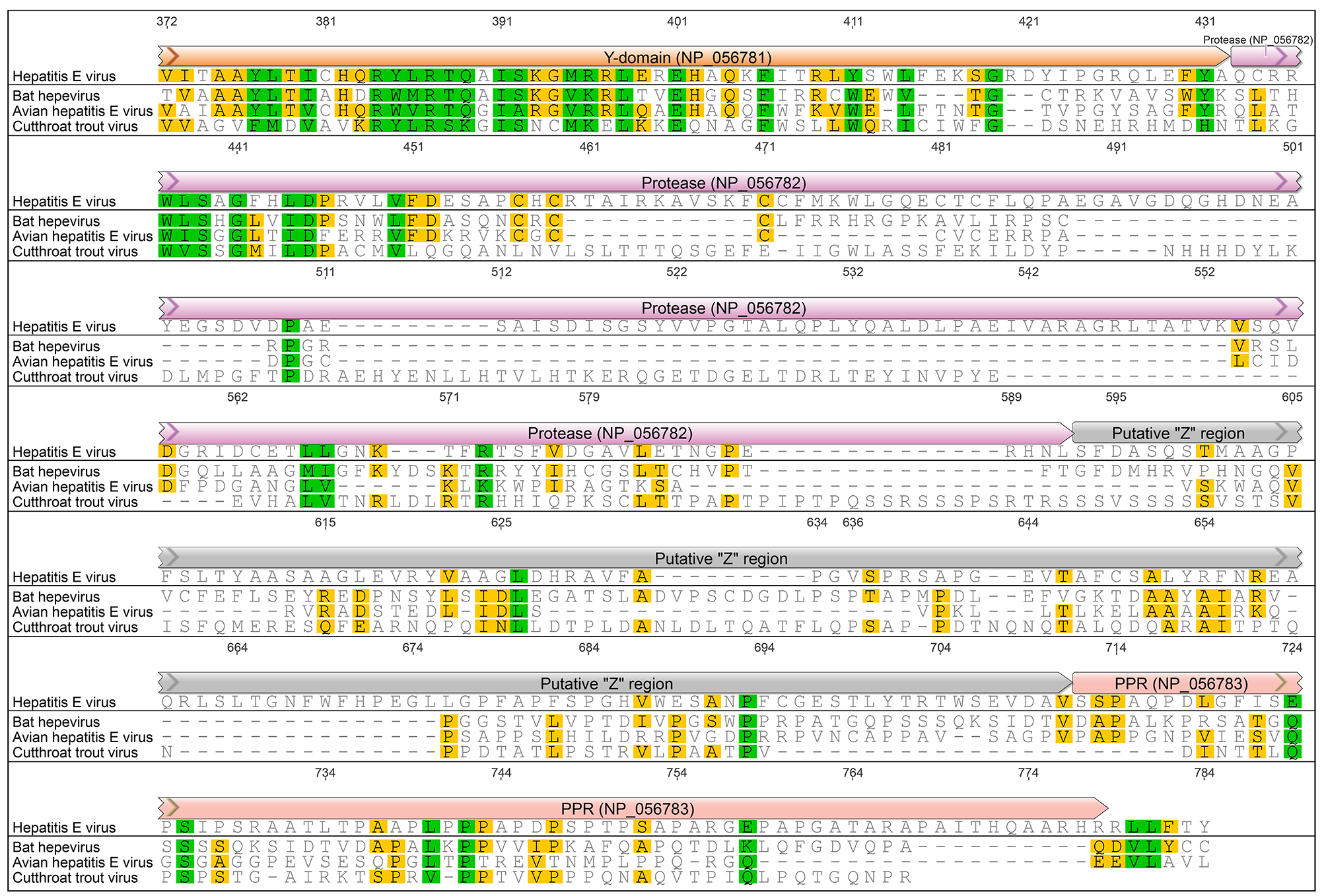

Supplement: Additional file 1: Figure S1. — Hepeviridae protease alignment. Alignment of the 3’ terminal of the Y-domain, protease domain and polyproline region of HEV GI and the three closest Hepeviridae homologues, using MAFFT. (TIF 2501 kb) [file 12862_2016_785_MOESM1_ESM.tif]

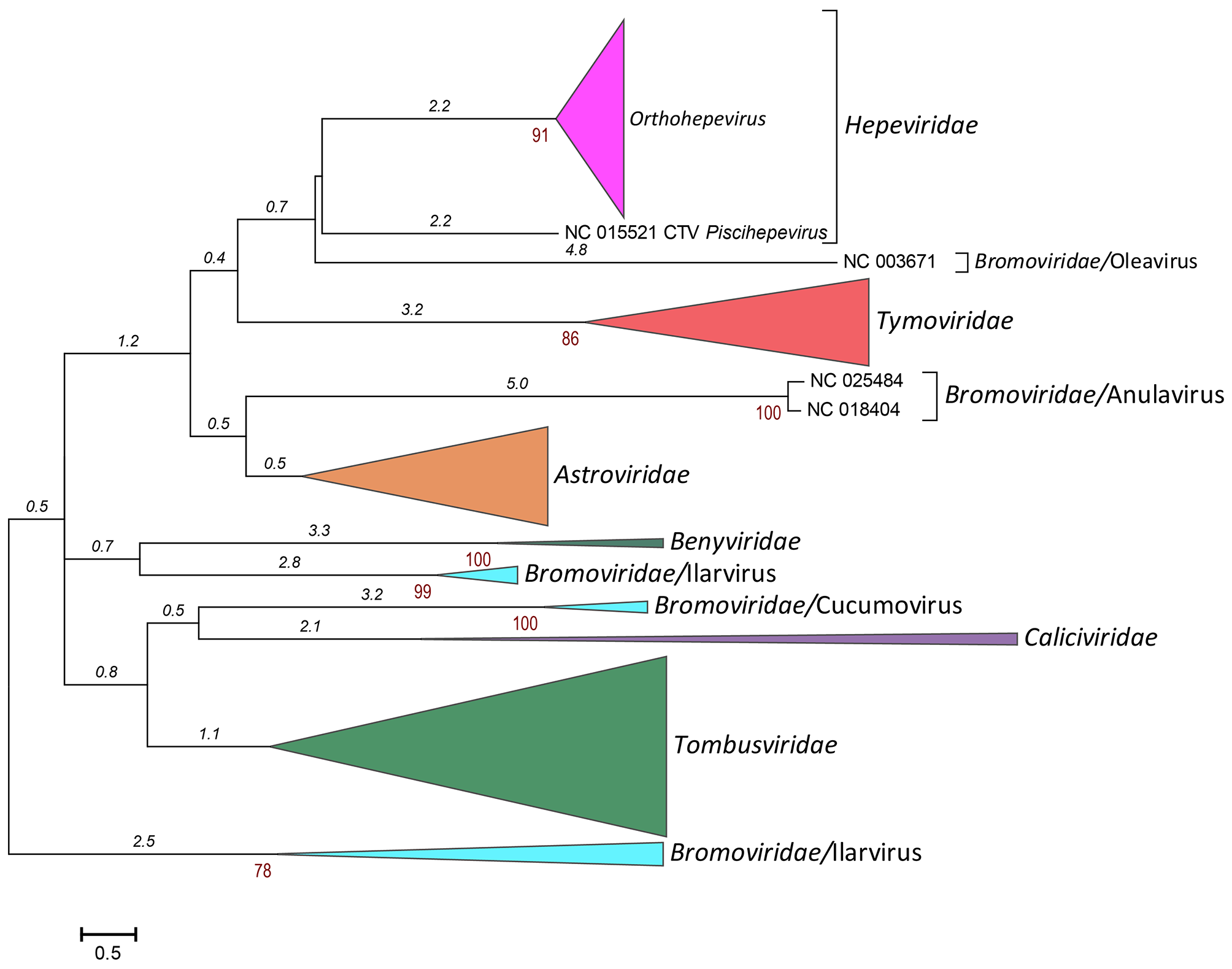

Supplement: Additional file 3: Figure S2. — Extended capsid phylogeny. Midpoint rooted maximum likelihood phylogenetic tree of 223 capsid sequences (Additional file 2: Table S3) from seven families, aligned by MAFFT and phylogeny performed using PhyML. Red values: bootstrap scores > 60 %; Black values: substitutions per site. (TIF 914 kb) [file 12862_2016_785_MOESM3_ESM.tif]
