# Supplementary material for: Ancient recombination events and the origins of hepatitis E virus
Source: BMC Evol Biol. 2016 Oct 12;16:210. doi: 10.1186/s12862-016-0785-y (PMC5062859; doi:10.1186/s12862-016-0785-y)
Supplement: Additional file 2: Table S1. — List of Hepeviridae genomes analyzed. Table S2. List of capsid and RdRp sequences used for Figs. 8 and 9. Table S3. List of 223 genomic sequences from capsid sequences used for creating additional Fig. 2. (DOCX 122 kb) [file 12862_2016_785_MOESM2_ESM.docx]

# Additional Files

**Additional Figure 1. *Hepeviridae* protease alignment.** Alignment of the 3’ terminal of the Y-domain, protease domain and polyproline region of *Orthohepeviridae* A HEV GI and the three closest *Hepeviridae* homologues, using MAFFT.

**Additional Figure 2. Extended capsid phylogeny.** Midpoint rooted maximum likelihood phylogenetic tree of 223 capsid sequences (S3 Table) from seven families, aligned by MAFFT and phylogeny performed using PhyML. Red values: bootstrap scores > 60 %; Black values: substitutions per site.

## Additional Table 1: List of *Hepeviridae* genomes analyzed.

| **Sequence Name** | **Length** | **Country of isolation** | **Collection date** | **Genotype** | **Host** | **Isolate** |
| --- | --- | --- | --- | --- | --- | --- |
| AB073912 | 7,257 | Japan | 2000 | 3b | *Sus scrofa* | swJ570 |
| AB074915 | 7,236 | Japan: Saitama | 1994 | 4c | *Homo sapiens* | JAK-Sai |
| AB074917 | 7,235 | Japan: Sapporo | 2000 | 4c | *Homo sapiens* | JKK-Sap |
| AB074918 | 7,256 | Japan: Sapporo | 2001 | 3a | *Homo sapiens* | JKN-Sap |
| AB074920 | 7,240 | Japan: Sapporo | 2001 | 3a | *Homo sapiens* | JMY-Haw |
| AB080575 | 7,186 | Japan | 2000 | 4c | *Homo sapiens* | HE-JI4 |
| AB089824 | 7,262 | Japan: Tokyo | 1993 | 3a | *Homo sapiens* | HE-JA10 |
| AB091394 | 7,218 | Japan: Kanagawa | 1998 | 3b | *Homo sapiens* | JJT-Kan |
| AB091395 | 7,234 | Japan: Hokkaido | 2002 | 4c | *Homo sapiens* | JSN-Sap-FH |
| AB097811 | 7,258 | Japan: Hokkaido | 2002 | 4c | *Sus scrofa* | swJ13-1 |
| AB097812 | 7,258 | Japan: Hokkaido | 1997 | 4c | *Homo sapiens* | HE-JA1 |
| AB099347 | 7,250 | Japan: Tochigi | 2002 | 4c | *Homo sapiens* | HE-JK4 |
| AB108537 | 7,193 | China: Changchun | 2000 | 4g | *Homo sapiens* | CCC220 |
| AB161717 | 7,202 | Japan: Hokkaido | 1995 | 4c | *Homo sapiens* | JSM-Sap95 |
| AB161718 | 7,202 | Japan: Hokkaido | 2002 | 4c | *Homo sapiens* | JTS-Sap02 |
| AB161719 | 7,202 | Japan: Hokkaido | 2002 | 4c | *Homo sapiens* | JYW-Sap02 |
| AB189070 | 7,247 | Japan: Hyogo | 2004 | 3b | *Sus scrofa* | JBOAR1-Hyo04 |
| AB189071 | 7,230 | Japan: Hyogo | 2003 | 3b | *Cervus nippon* | JDEER-Hyo03L |
| AB189072 | 7,180 | Japan: Hyogo | 2003 | 3b | *Homo sapiens* | JMO-Hyo03L |
| AB189073 | 7,180 | Japan: Hyogo | 2003 | 3b | *Homo sapiens* | JSO-Hyo03L |
| AB189074 | 7,180 | Japan: Hyogo | 2003 | 3b | *Homo sapiens* | JTH-Hyo03L |
| AB189075 | 7,180 | Japan: Hyogo | 2003 | 3b | *Homo sapiens* | JYO-Hyo03L |
| AB193176 | 7,251 | Japan: Tottori | 2003 | 4c | *Homo sapiens* | JSF-Tot03C |
| AB193177 | 7,256 | Japan: Hokkaido | 2001 | 4c | *Homo sapiens* | JYN-Sap01C |
| AB193178 | 7,154 | Japan: Niigata | 2002 | 4c | *Homo sapiens* | JYN-Nii02L |
| AB197673 | 7,257 | China: Xian | 1998 | 4a | *Homo sapiens* | JKO-ChiSai98C |
| AB197674 | 7,260 | China: Shanghai | 2001 | 4a | *Homo sapiens* | JYI-ChiSai01C |
| AB200239 | 7,251 | Japan: Hokkaido | 2002 | 4c | *Homo sapiens* | JSN-Sap-FH02C |
| AB220971 | 7,262 | Japan: Mito | 1998 | 4 | *Homo sapiens* | HE-JF3 |
| AB220972 | 7,271 | Japan: Hokkaido | 2002 | 4c | *Homo sapiens* | HE-JF4 |
| AB220973 | 7,270 | Japan: Hokkaido | 2002 | 4c | *Homo sapiens* | HE-JF5 |
| AB220974 | 7,268 | Japan: Hokkaido | 1998 | 4f | *Homo sapiens* | HE-JA2 |
| AB220975 | 7,262 | Japan: Hokkaido | 2002 | 4c | *Homo sapiens* | HE-JA19 |
| AB220976 | 7,266 | Japan: Hokkaido | 2002 | 4c | *Homo sapiens* | HE-JA28 |
| AB220977 | 7,266 | Japan: Hokkaido | 2003 | 4c | *Homo sapiens* | HE-JA36 |
| AB220978 | 7,281 | Japan: Hokkaido | 2004 | 4c | *Homo sapiens* | HE-JA37 |
| AB220979 | 7,265 | Japan: Hokkaido | 2004 | 4c | *Homo sapiens* | HE-JA41 |
| AB222182 | 7,240 | Japan: Saga | 2005 | 3b | *Sus scrofa* | wbJSG1 |
| AB222183 | 7,241 | Japan: Tokushima | 2005 | 3b | *Sus scrofa* | wbJTS1 |
| AB222184 | 7,240 | Japan: Yamaguchi | 2005 | 3b | *Sus scrofa* | wbJYG1 |
| AB236320 | 7,236 | Japan: Okinawa | 2002 | 3b | *Herpestes javanicus* | JMNG-Oki02C |
| AB246676 | 7,231 | Japan | 2005 | 3b | *Homo sapiens* | HEVN1 |
| AB248520 | 7,280 | Japan: Mie | 2004 | 3e | *Homo sapiens* | HE-JA04-1911 |
| AB248521 | 7,241 | Japan: Hokkaido | 2001 | 3e | *Sus scrofa* | swJ8-5 |
| AB248522 | 7,241 | Japan: Hokkaido | 2001 | 3e | *Sus scrofa* | swJ12-4 |
| AB253420 | 7,253 | Japan: Okinawa | 2005 | 4b | *Homo sapiens* | HEVN2 |
| AB290312 | 7,237 | Mongolia | 2006 | 3 | *Sus scrofa* | swMN06-A1288 |
| AB290313 | 7,239 | Mongolia | 2006 | 3 | *Sus scrofa* | swMN06-C1056 |
| AB291951 | 7,215 | Japan: Saitama | 1997 | 3 | *Homo sapiens* | JIO-Sai97L |
| AB291952 | 7,215 | Japan: Tottori | 2005 | 3 | *Homo sapiens* | JIY-Tot05L |
| AB291953 | 7,215 | Japan: Okinawa | 2005 | 3 | *Homo sapiens* | JSO-Oki05L |
| AB291954 | 7,215 | Japan: Okayama | 2004 | 3 | *Homo sapiens* | JSS-Oka04L |
| AB291955 | 7,215 | Japan: Kyoto | 2006 | 3b | *Homo sapiens* | JSW-Kyo-FH06L |
| AB291956 | 7,215 | Japan: Tottori | 2004 | 3 | *Homo sapiens* | JYM-Tot04L |
| AB291957 | 7,215 | Japan: Okinawa | 2004 | 3 | *Homo sapiens* | JYU-Oki04L |
| AB291958 | 7,168 | Japan: Ehime | 2004 | 3e | *Homo sapiens* | JNH-Ehi04L |
| AB291959 | 7,209 | Japan: Hokkaido | 2004 | 4 | *Homo sapiens* | JTC-Kit-FH04L |
| AB291960 | 7,236 | Japan: Kagawa | 2006 | 3 | *Homo sapiens* | JTK-Kag06C |
| AB291961 | 7,241 | Japan: Osaka | 2004 | 3f | *Homo sapiens* | JMH-Osa04C |
| AB291962 | 7,236 | Japan: Toyama | 2004 | 3b | *Homo sapiens* | JHK-Toy04C |
| AB291963 | 7,236 | Japan: Toyama | 2005 | 3b | *Homo sapiens* | JRM-Toy05C |
| AB291964 | 7,244 | Japan: Tokyo | 2003 | 4b | *Homo sapiens* | JYK-Tok03C |
| AB291965 | 7,255 | Japan: Hokkaido | 2004 | 4 | *Homo sapiens* | HRC-HE14C |
| AB291966 | 7,255 | Japan: Hokkaido | 2004 | 4 | *Homo sapiens* | JST-KitAsa04C |
| AB291967 | 7,255 | Japan: Hokkaido | 2006 | 4 | *Homo sapiens* | JKO-Aba-FH06C |
| AB291968 | 7,255 | Japan: Hokkaido | 2006 | 4 | *Homo sapiens* | JMM-Aba06C |
| AB301710 | 7,246 | Japan | 2003 | 3b | *Homo sapiens* | JE03-1760F |
| AB369687 | 7,217 | Japan: Kanagawa | 1998 | 3f | *Homo sapiens* | E116-YKH98C |
| AB369688 | 7,227 | Japan: Hokkaido | 2004 | 4 | *Homo sapiens* | E087-SAP04C |
| AB369689 | 7,215 | Japan: Saitama | 2004 | 3 | *Homo sapiens* | E088-STM04C |
| AB369690 | 7,236 | Japan: Tokyo | 2005 | 4i | *Homo sapiens* | E067-SIJ05C |
| AB369691 | 7,216 | Japan: Osaka | 2005 | 3b | *Homo sapiens* | E097-OSA05C |
| AB443623 | 7,210 | Japan: Miyazaki | 2002 | 3 | *Sus scrofa* | JIO-swJ19-1 |
| AB443624 | 7,210 | Japan: Miyazaki | 2002 | 3 | *Sus scrofa* | JIO-swJ19-2 |
| AB443625 | 7,210 | Japan: Miyazaki | 2002 | 3 | *Sus scrofa* | JIO-swJ19-5 |
| AB443626 | 7,210 | Japan: Miyazaki | 2002 | 3 | *Sus scrofa* | JIO-swJ19-7 |
| AB443627 | 7,210 | Japan: Miyazaki | 2002 | 3 | *Sus scrofa* | JIO-swJ19-8 |
| AB481226 | 7,239 | Japan | 2008 | 3 | *Sus scrofa* | swJB-E10 |
| AB481227 | 7,253 | Japan | 2008 | 4c | *Sus scrofa* | swJB-H7 |
| AB481228 | 7,267 | Japan | 2008 | 3a | *Sus scrofa* | swJB-M8 |
| AB481229 | 7,239 | Japan | 2008 | 3b | *Sus scrofa* | swJR-P5 |
| AB521805 | 7,219 | Japan: Hamamatsu | 2007 | 4i | *Homo sapiens* | JKS-Shiz07L |
| AB521806 | 7,219 | Japan: Hamamatsu | 2008 | 4i | *Homo sapiens* | JYN-Shiz08L |
| AB573435 | 7,267 | Japan: Shizuoka | 2009 | 5a | *Sus scrofa* | JBOAR135-Shiz09 |
| AB591733 | 7,236 | Japan: Okinawa | 2008 | 3a | *Herpestes javanicus* | JMNG26-Oki08 |
| AB591734 | 7,249 | Japan: Okinawa | 2008 | 3a | *Herpestes javanicus* | JMNG36-Oki08 |
| AB602439 | 7,252 | Japan: Aichi | 2005 | 4 | *Homo sapiens* | HE-Aichi-C1 |
| AB602440 | 7,252 | Japan: Gifu | 2008 | 4i | *Sus scrofa* | wbJGF_08-1 |
| AB602441 | 7,261 | Japan: Okayama | 2006 | 6a | *Sus scrofa* | wbJOY_06 |
| AB630970 | 7,256 | Japan: Hokkaido | 2007 | 3a | *Homo sapiens* | HRC-HE104 |
| AB630971 | 7,238 | Japan: Fukuoka | 2003 | 3b | *Homo sapiens* | JRC-HE3 |
| AB698071 | 7,226 | Japan: Tsukuba | 2000 | 3b | *Sus scrofa* | Highland/2000 |
| AB698654 | 7,236 | Japan: Yamaguchi | 2011 | 4g | *Homo sapiens* | JTF-Yamagu11 |
| AB720034 | 7,186 | Bangladesh: Rajshahi | 2010 | 1a | *Homo sapiens* | E11-Ban10 |
| AB720035 | 7,186 | Bangladesh: Rajshahi | 2010 | 1f | *Homo sapiens* | E13-Ban10 |
| AB740220 | 7,300 | Inner Mongolia | 2011 | 3ra | *Oryctolagus cuniculus* | rbIM199 |
| AB740221 | 7,305 | Inner Mongolia | 2011 | 3ra | *Oryctolagus cuniculus* | rbIM022 |
| AB740222 | 7,307 | Inner Mongolia | 2011 | 3ra | *Oryctolagus cuniculus* | rbIM004 |
| AB740232 | 7,266 | Japan | 2010 | 3 | *Sus scrofa* | G3-HEV83-2-27 |
| AB780450 | 7,111 | Japan: Mie | 2010 | 3e | *Sus scrofa* | JBOAR100-Mie10 |
| AB780451 | 7,139 | Japan: Mie | 2011 | 3e | *Sus scrofa* | JBOAR107-Mie11 |
| AB780452 | 7,165 | Japan: Mie | 2011 | 3e | *Sus scrofa* | JBOAR111-Mie11 |
| AB780453 | 7,151 | Japan: Mie | 2011 | 3e | *Sus scrofa* | JBOAR124-Mie11 |
| AB856243 | 7,263 | Japan: Nagano | 2013 | 6 | *Sus scrofa* | wbJNN_13 |
| AB909124 | 7,214 | Japan: Gifu | 2012 | 4i | *Homo sapiens* | JAO-Gif12 |
| AB909125 | 7,214 | Japan: Shizuoka | 2013 | 4i | *Homo sapiens* | JKK-Shiz13 |
| AF051830 | 7,199 | Nepal | 1992 | 1a | *Homo sapiens* | TK15/92 |
| AF060668 | 7,202 | USA | 1995 | 3a | *Homo sapiens* | HEV-US1 |
| AF060669 | 7,277 | USA | 1996 | 3a | *Homo sapiens* | HEV-US2 |
| AF076239 | 7,194 | India: Hyderabad | 1990 | 1a | *Homo sapiens* | Hyderabad |
| AF082843 | 7,207 | USA | 1996 | 3a | *Sus scrofa* | Meng |
| AF185822 | 7,143 | Pakistan | 1988 | 1a | *Homo sapiens* | Abb-2B |
| AF455784 | 7,239 | Kyrgyzstan: Osh | 1987 | 3g | *Homo sapiens* | Osh 205 |
| AF459438 | 7,206 | India: Yamuna Nagar | 1989 | 1a | *Homo sapiens* | Yam67/89 |
| AJ272108 | 7,232 | China | 1999 | 4d | *Homo sapiens* | T1 |
| AP003430 | 7,230 | Japan | 2000 | 3b | *Homo sapiens* | JRA1 |
| AY115488 | 7,255 | Canada: Ontario | 2001 | 3j | *Sus scrofa* | Arkell |
| AY204877 | 7,170 | Chad | 1984 | 1e | *Homo sapiens* | T3 |
| AY230202 | 7,212 | Morocco | 2002 | 1d | *Homo sapiens* | Morocco |
| AY594199 | 7,270 | China :Xinjian | 2001 | 4d | *Sus scrofa* | swCH25 |
| AY723745 | 7,262 | India | 2000 | 4e | *Sus scrofa* | IND-SW-00-01 |
| D10330 | 7,194 | Burma | 1986 | 1a | *Homo sapiens* | Bur86 |
| D11092 | 7,207 | China: Xinjiang | 1987 | 1b | *Homo sapiens* | Hetian88 |
| D11093 | 7,194 | China: Xinjiang | 1987 | 1b | *Homo sapiens* | Uigh179 |
| DQ279091 | 7,234 | China | 2005 | 4b | *Sus scrofa* | swDQ |
| DQ450072 | 7,215 | India: western region | 2006 | 4i | *Homo sapiens* | swCH31 |
| DQ459342 | 7,261 | India | 2000 | 1a | *Homo sapiens* |  |
| EF077630 | 7,293 | China | 2006 | 4a | *Sus scrofa* | Ch-S-1 |
| EF570133 | 7,258 | China | 2006 | 4i | *Sus scrofa* | SH-SW-zs1 |
| EU360977 | 7,281 | Sweden | 2006 | 3 | *Sus scrofa* | swX07-E1 |
| EU366959 | 7,245 | China | 2006 | 4a | *Sus scrofa* | swGX32 |
| EU375463 | 7,321 | Thailand | 2006 | 3f | *Homo sapiens* | Thai-swHEV07 |
| EU495148 | 7,269 | France | 2008 | 3f | *Homo sapiens* | TLS25 |
| EU676172 | 7,216 | China | 2007 | 4b | *Sus scrofa* | swGX40 |
| EU723512 | 7,192 | Spain | 2007 | 3f | *Sus scrofa* | SW626 |
| EU723513 | 7,304 | Spain | 2007 | 3f | *Sus scrofa* | SW627 |
| EU723514 | 7,279 | Spain | 2007 | 3f | *Sus scrofa* | SWP6 |
| EU723515 | 7,279 | Spain | 2007 | 3f | *Sus scrofa* | SWP7 |
| EU723516 | 7,251 | Spain | 2007 | 3f | *Sus scrofa* | SWP8 |
| FJ426403 | 7,239 | South Korea | 2007 | 3a | *Sus scrofa* | swKOR-1 |
| FJ426404 | 7,217 | South Korea | 2007 | 3a | *Sus scrofa* | swKOR-2 |
| FJ457024 | 7,255 | India | 2005 | 1 | *Homo sapiens* | HEV-H |
| FJ527832 | 7,284 | China: Shanghai | 2008 | 3b | *Sus scrofa* | SAAS-JDY5 |
| FJ610232 | 7,216 | China: Gansu | 2008 | 4d | *Sus scrofa* | swCH189 |
| FJ653660 | 7,237 | Thailand | 2008 | 3f | *Homo sapiens* | CU001 |
| FJ705359 | 7,260 | Germany | 2006 | 3c | *Sus scrofa* | wbGER27 |
| FJ763142 | 7,318 | South Korea | 2007 | 4a | *Homo sapiens* | KNIH-hHEV4 |
| FJ906895 | 7,301 | China | 2008 | 3ra | *Oryctolagus cuniculus* | GDC9 |
| FJ906896 | 7,246 | China | 2008 | 3ra | *Oryctolagus cuniculus* | GDC46 |
| FJ956757 | 7,206 | Germany | 2005 | 3f | *Homo sapiens* | HEV_RKI |
| FJ998008 | 7,153 | Germany: Brandenburg | 2007 | 3i | *Sus scrofa* | BB02 |
| FJ998015 | 7,258 | Germany: Brandenburg | 2007 | 3e | *Sus scrofa* | SA21 |
| GU119960 | 7,264 | China | 2009 | 4a | *Sus scrofa* | CHN-XJ-SW33 |
| GU119961 | 7,251 | China | 2009 | 4h | *Sus scrofa* | CHN-XJ-SW13 |
| GU188851 | 7,270 | China: Wuhan | 2009 | 4h | *Sus scrofa* | WH09 |
| GU206559 | 7,250 | China | 2008 | 4d | *Sus scrofa* | bjsw1 |
| GU361892 | 7,310 | China | 2008 | 4d | *Sus scrofa* | hb-3 |
| GU937805 | 7,189 | China | 2009 | 3ra | *Oryctolagus cuniculus* | ch-bj-n1 |
| HM055578 | 7,272 | Hungary | 2005 | 3e | *Sus scrofa* | HEV072/sw/HUN-05 |
| HM152568 | 7,229 | China | 2008 | 4d | *Sus scrofa* | bisw5 |
| HM439284 | 7,264 | China | 2008 | 4i | *Homo sapiens* | EChZ20 |
| HQ389543 | 7,256 | United Kingdom | 2009 | 3 | *Homo sapiens* | Kernow-C1 |
| HQ634346 | 7,206 | Taiwan | 1998 | 4a | *Homo sapiens* | TW6196E |
| JF443717 | 7,215 | India: Kolhapur | 1991 | 1c | *Homo sapiens* | HEV-AVH1-1991 |
| JF443718 | 7,215 | India: Pune | 1998 | 1 | *Homo sapiens* | HEV-AVH2-1998 |
| JF443719 | 7,206 | India: Lonavala | 2000 | 1a | *Homo sapiens* | HEV-AVH3-2000 |
| JF443720 | 7,217 | India: Pune | 2006 | 1a | *Homo sapiens* | HEV-AVH4-2006 |
| JF443721 | 7,206 | India: Roha | 2010 | 1f | *Homo sapiens* | HEV-AVH5-2010 |
| JF443722 | 7,211 | India: Pune | 2003 | 1f | *Homo sapiens* | HEV-FHF1-2003 |
| JF443723 | 7,206 | India: Pune | 2004 | 1f | *Homo sapiens* | HEV-FHF2-2004 |
| JF443724 | 7,201 | India: Pune | 2005 | 1f | *Homo sapiens* | HEV-FHF3-2005 |
| JF443725 | 7,226 | India: Pune | 2006 | 1f | *Homo sapiens* | HEV-FHF4-2006 |
| JF443726 | 7,262 | India: Pune | 2007 | 1f | *Homo sapiens* | HEV-FHF5-2007 |
| JF915746 | 7,314 | China | 2009 | 4i | *Sus scrofa* | SAAS-FX17 |
| JN564006 | 7,251 | USA | 2010 | 3a | *Homo sapiens* | LBPR-000379 |
| JN837481 | 7,261 | USA | 2008 | 3a | *Homo sapiens* | US-C031008 |
| JN906974 | 7,259 | France | 2010 | 3f | *Homo sapiens* | FR-HuHEVF3f |
| JN906975 | 7,317 | France | 2010 | 3f | *Sus scrofa* | FR-SHEVF3f |
| JN906976 | 7,271 | France | 2010 | 3f | *Sus scrofa* | FR-SHEVB3f |
| JQ013791 | 7,238 | France | 2007 | 3ra | *Oryctolagus cuniculus* | W1-11 |
| JQ013792 | 7,281 | France | 2007 | 3ra | *Oryctolagus cuniculus* | W7-57 |
| JQ013793 | 7,182 | France | 2008 | 3ra | *Homo sapiens* | TLS-18516 |
| JQ013794 | 7,233 | France | 2007 | 3h | *Homo sapiens* | TR19 |
| JQ013795 | 7,212 | France | 2006 | 3e | *Homo sapiens* | TR02 |
| JQ026407 | 7,291 | Japan: Inuyama | 2009 | 3e | *Macaca fuscata* | Inuyama |
| JQ655733 | 7,212 | China | 2006 | 4a | *Homo sapiens* | MO |
| JQ655734 | 7,257 | China | 2006 | 1b | *Homo sapiens* | W2-1 |
| JQ655735 | 7,261 | China | 2006 | 4h | *Homo sapiens* | W3 |
| JQ655736 | 7,252 | China | 2006 | 4d | *Sus scrofa* | W2-5 |
| JQ740781 | 7,296 | China | 2009 | 4h | *Homo sapiens* | CHN-NJ-H2011 |
| JQ768461 | 7,238 | China | 2011 |  | *Oryctolagus cuniculus* | CHN-BJ-rb14 |
| JQ953664 | 7,244 | France | 2006 | 3 | *Sus scrofa* | FR-SHEV3c-like |
| JQ953665 | 7,249 | France | 2006 | 3e | *Sus scrofa* | FR-SHEV3e |
| JQ953666 | 7,244 | France | 2008 | 3f | *Sus scrofa* | FR-SHEV3f |
| JQ993308 | 7,296 | China | 2009 | 4i | *Sus scrofa* | HEV-ZJ1 |
| JX109834 | 7,296 | China | 2011 |  | *Oryctolagus cuniculus* | CHN-BJ-R14 |
| JX121233 | 7,306 | China | 2011 |  | *Oryctolagus cuniculus* | CHN-BJ-R14 |
| JX565469 | 7,233 | USA: Virginia | 2010 | 3ra | *Oryctolagus cuniculus* | CMC-1 |
| JX855794 | 7,253 | China | 2011 | 4b | *Sus scrofa* | SS19 |
| KC163335 | 7,306 | China | 2012 | 4d | *Homo sapiens* | CH-YT-1 |
| KC166967 | 7,313 | France | 2004 | 3f | *Homo sapiens* | TLS09-0 |
| KC166968 | 7,313 | France | 2005 | 3f | *Homo sapiens* | TLS09-1 |
| KC166969 | 7,314 | France | 2006 | 3f | *Homo sapiens* | TLS09-2 |
| KC166970 | 7,388 | France | 2007 | 3f | *Homo sapiens* | TLS09-3 |
| KC166971 | 7,249 | France | 2008 | 3f | *Homo sapiens* | TLS09-4 |
| KC492825 | 7,423 | China | 2011 | 4a | *Homo sapiens* | CH-YT-HEV02 |
| KC618402 | 7,301 | Germany | 2011 | 3c | *Homo sapiens* | 47832 |
| KC692453 | 7,249 | China | 2011 | 4a | *Sus scrofa* | CH-YT-sHEV01 |
| KF176351 | 7,141 | China | 2011 | 4d | *Sus scrofa* | CHN-SD-sHEV |
| KF303502 | 7,234 | Germany | 2006 | 3a | *Sus scrofa* | GiSw |
| KF736234 | 7,132 | China | 2013 | 4 | *Bos grunniens* | CHN-QH-Yak27 |
| KF922359 | 7,296 | France | 2009 | 3e | *Homo sapiens* | rt93100008 |
| KJ013414 | 7,296 | China | 2013 |  | *Oryctolagus cuniculus* | CHN-BJ-r14(8) |
| KJ013415 | 7,264 | China | 2013 | 3 | *Oryctolagus cuniculus* | CHN-BJ-r14(9) |
| KJ155502 | 7,224 | China | 2010 | 4h | *Sus scrofa* | KM01 |
| KJ496143 | 7,223 | United Arab Emirates | 2013 | 7a | *Camelus dromedarius* | 178C |
| KJ496144 | 7,243 | United Arab Emirates | 2013 | 7 | *Camelus dromedarius* | 180C |
| KJ507955 | 7,225 | Canada | 2003 | 3a | *Sus scrofa* | swSTHY12-VAS19/2003/CA |
| KJ507956 | 7,110 | Canada | 2003 | 3b | *Sus scrofa* | swSTHY42-VAS49/2003/CA |
| KJ701409 | 7,157 | France | 2009 | 3c | *Homo sapiens* | AB_7110 |
| L25547 | 7,221 | China | 1987 | 1 | *Homo sapiens* | HPEGENA |
| L25595 | 7,207 | China | 1987 | 1b | *Homo sapiens* | KS2-87 |
| L08816 | 7,202 | China | 1987 | 1b | *Homo sapiens* | HeBei |
| M73218 | 7,180 | Burma | 1982 | 1a | *Homo sapiens* | Bur82 |
| M74506 | 7,138 | Mexico | 1986 | 2a | *Homo sapiens* | Mexican (Mex-14) |
| M80581 | 7,200 | Pakistan | 1987 | 1b | *Homo sapiens* | SAR-55 |
| M94177 | 7,176 | China | 1987 | 1b | *Homo sapiens* | China |
| NC001434 | 7,202 | China | 1988 | 1b | *Homo sapiens* | HeBei |
| X98292 | 7,194 | India | 1992 | 1c | *Homo sapiens* | HEV037 |
| X99441 | 7,194 | India | 1993 | 1a | *Homo sapiens* | Madras |

## Additional Table 2: List of capsid and RdRp sequences used for Figures 8 and 9.

| **Species** | **Order** | **Family** | **Genus** | **Capsid Accession** | **RdRp Accession** |
| --- | --- | --- | --- | --- | --- |
| Equine arteritis virus | *Nidovirales* | *Arteriviridae* | Arterivirus | NP705587 | NP127506 |
| Feline infectious peritonitis virus | *Nidovirales* | *Coronaviridae* | Alphacoronavirus | YP004070198 | YP004070193 |
| White bream virus | *Nidovirales* | *Coronaviridae* | Bafinivirus | YP803216 | YP803213 |
| Murine hepatitis virus | *Nidovirales* | *Coronaviridae* | Betacoronavirus | NP045301 | NP740616 |
| Thrush coronavirus | *Nidovirales* | *Coronaviridae* | Deltacoronavirus | YP002308499 | YP002308496 |
| Infectious bronchitis virus | *Nidovirales* | *Coronaviridae* | Gammacoronavirus | NP040835 | NP740629 |
| Berne virus | *Nidovirales* | *Coronaviridae* | Torovirus | P27904 | P0C6V7 |
| Cavally virus | *Nidovirales* | *Mesoniviridae* | Alphamesonivirus | AEH26448 | YP00459898 |
| Gill-associated virus | *Nidovirales* | *Roniviridae* | Okavirus | YP001661454 | YP001661452 |
| Acute bee paralysis virus | *Picornavirales* | *Dicistroviridae* | Aparavirus | NP066242 | NP066241 |
| Cricket paralysis virus | *Picornavirales* | *Dicistroviridae* | Cripavirus | NP647482 | NP647481 |
| Foot-and-mouth disease virus | *Picornavirales* | *Picornaviridae* | Aphthovirus | NP740458 | NP658990 |
| Seal picornavirus | *Picornavirales* | *Picornaviridae* | Aquamavirus | YP001497152 | YP001497152 |
| Duck hepatitis A virus | *Picornavirales* | *Picornaviridae* | Avihepatovirus | YP007947990 | YP007947990 |
| Turkey avisivirus | *Picornavirales* | *Picornaviridae* | Avisivirus | AGF33849 | AGF33849 |
| Cherry rasp leaf virus | *Picornavirales* | *Secoviridae* | Cheravirus | YP081445 | YP081444 |
| Cowpea mosaic virus | *Picornavirales* | *Secoviridae* | Comovirus | NP613286 | NP613283 |
| Satsuma dwarf virus | *Picornavirales* | *Secoviridae* | Sadwavirus | BAD12076 | NP620566 |
| Parsnip yellow fleck virus | *Picornavirales* | *Secoviridae* | Sequivirus | NP619734 | NP619734 |
| Tomato torrado virus | *Picornavirales* | *Secoviridae* | Torradovirus | YP001040018 | YP001039627 |
| Rice tungro spherical virus | *Picornavirales* | *Secoviridae* | Waikavirus | NP042507 | NP042507 |
| Shallot virus X | *Tymovirales* | *Alphaflexiviridae* | Allexivirus | NP620652 | Q04575 |
| Botrytis virus X | *Tymovirales* | *Alphaflexiviridae* | Botrexvirus | NP932309 | NP932306 |
| Lolium latent virus | *Tymovirales* | *Alphaflexiviridae* | Lolavirus | YP001718503 | YP001718499 |
| Indian citrus ringspot virus | *Tymovirales* | *Alphaflexiviridae* | Mandarivirus | NP203557 | NP203553 |
| Narcissus mosaic virus | *Tymovirales* | *Alphaflexiviridae* | Potexvirus | NP040782 | NP040778 |
| Grapevine virus A | *Tymovirales* | *Betaflexiviridae* | Vitivirus | NP619665 | NP619662 |
| Apple stem grooving virus | *Tymovirales* | *Betaflexiviridae* | Capillovirus | NP044335 | NP044335 |
| **Species** | **Order** | **Family** | **Genus** | **Capsid Accession** | **RdRp Accession** |
| Potato virus M | *Tymovirales* | *Betaflexiviridae* | Carlavirus | NP056771 | NP056767 |
| Apple stem pitting virus | *Tymovirales* | *Betaflexiviridae* | Foveavirus | NP604468 | NP604464 |
| Potato virus T | *Tymovirales* | *Betaflexiviridae* | Tepovirus | YP002019750 | YP002019748 |
| Apple chlorotic leaf spot virus | *Tymovirales* | *Betaflexiviridae* | Trichovirus | NP040553 | NP040551 |
| Botrytis virus F | *Tymovirales* | *Gammaflexiviridae* | Mycoflexivirus | NP068550 | NP068549 |
| Grapevine fleck virus | *Tymovirales* | *Tymoviridae* | Maculavirus | NP542613 | NP542612 |
| Maize rayado fino virus | *Tymovirales* | *Tymoviridae* | Marafivirus | NP734077 | NP734076 |
| Turnip yellow mosaic virus | *Tymovirales* | *Tymoviridae* | Tymovirus | NP663298 | NP663297 |
| Nudaurelia capensis beta virus |  | *Alphatetraviridae* | Betatetravirus | NP048060 | NP048059 |
| Helicoverpa armigera stunt virus |  | *Alphatetraviridae* | Omegatetravirus | NP049237 | NP049235 |
| Avastrovirus |  | *Astroviridae* | Avastrovirus | CAB95007 | CAB95006 |
| Chicken astrovirus |  | *Astroviridae* | Avastrovirus | AEE88305 | AEE88304 |
| Duck astrovirus |  | *Astroviridae* | Avastrovirus | YP002728003 | YP002728002 |
| Human astrovirus |  | *Astroviridae* | Mamastrovirus | CAA81032 | CAA81033 |
| Mushroom bacilliform virus |  | *Barnaviridae* | Barnavirus | NP042511 | NP042510 |
| Beet necrotic yellow vein virus |  | *Benyviridae* | Benyvirus | NP705610 | NP705701 |
| Beet soil-borne mosaic virus |  | *Benyviridae* | Benyvirus | NP612588 | AF280539 |
| Alfalfa mosaic virus |  | *Bromoviridae* | Alfamovirus | NP041195 | YP053235 |
| Pelargonium zonate spot virus |  | *Bromoviridae* | Anulavirus | NP619773 | NP619771 |
| Brome mosaic virus |  | *Bromoviridae* | Bromovirus | NP041199 | NP041197 |
| Cucumber mosaic virus |  | *Bromoviridae* | Cucumovirus | NP040777 | NP049324 |
| Tobacco streak virus |  | *Bromoviridae* | Ilarvirus | NP620774 | NP620768 |
| Olive latent virus |  | *Bromoviridae* | Oleavirus | NP620039 | NP620043 |
| Rabbit hemorrhagic disease virus |  | *Caliciviridae* | Lagovirus | NP740333 | NP062875 |
| Newbury -1 virus |  | *Caliciviridae* | Nebovirus | YP529550 | YP529550 |
| Norwalk virus |  | *Caliciviridae* | Norovirus | NP056821 | NP056820 |
| Sapovirus |  | *Caliciviridae* | Sapovirus | YP077278 | YP077278 |
| Vesicular exanthema of swine virus |  | *Caliciviridae* | Vesivirus | NP066256 | NP066255 |
| Beet yellows virus |  | *Closteroviridae* | Closterovirus | NP041874_5 | NP733949 |
| Lettuce infectious yellows virus |  | *Closteroviridae* | Crinivirus | NP619698 | NP619692 |
| Grapevine leafroll-associated virus |  | *Closteroviridae* | Velarivirus | YP004935925 | YP004935919 |
| Hepatitis C virus |  | *Flaviviridae* | Hepacivirus | NP751919 | NP671491 |
| Pegivirus A |  | *Flaviviridae* | Pegivirus | NP830210 | NP803209 |
| Bovine viral diarrhea virus |  | *Flaviviridae* | Pestivirus | NP776260 | NP776271 |
| Yellow fever virus |  | *Flaviviridae* | Yellow fever virus | NP775999 | NP776009 |
| Yellow fever virus |  | *Flaviviridae* | Yellow fever virus | NP740305 | NP776009 |
| Hepatitis E virus |  | *Hepeviridae* | Orthohepevirus | NP056788 | NP056779 |
| Cutthroat trout virus |  | *Hepeviridae* | Piscihepevirus | YP004464918 | YP004464929 |
| Enterobacteria phage Qbeta |  | *Leviviridae* | Allolevivirus | NP046750 | NP046752 |
| Enterobacteria phage MS2 |  | *Leviviridae* | Levivirus | NP040648 | NP040650 |
| **Species** | **Order** | **Family** | **Genus** | **Capsid Accession** | **RdRp Accession** |
| Pea enation mosaic virus |  | *Luteoviridae* | Enamovirus | NP620027 | NP620026 |
| Barley yellow dwarf virus |  | *Luteoviridae* | Luteovirus | NP840017 | NP840014 |
| Potato leafroll virus |  | *Luteoviridae* | Polerovirus | NP056751 | NP056748 |
| Nodamura virus |  | *Nodaviridae* | Alphanodavirus | NP077732 | NP077730 |
| Striped Jack nervous necrosis virus |  | *Nodaviridae* | Betanodavirus | NP599249 | NP599247 |
| Blackberry virus Y |  | *Potyviridae* | Brambyvirus | YP851208 | YP851006 |
| Barley yellow mosaic virus |  | *Potyviridae* | Bymovirus | NP734308 | NP148999 |
| Sweet potato mild mottle virus |  | *Potyviridae* | Ipomovirus | NP620656 | NP620656 |
| Triticum mosaic virus |  | *Potyviridae* | Poacevirus | YP002956096 | YP002956073 |
| Potato virus Y |  | *Potyviridae* | Potyvirus | NP734250 | NP056759 |
| Ryegrass mosaic virus |  | *Potyviridae* | Rymovirus | NP734328 | NP044727 |
| Wheat streak mosaic virus |  | *Potyviridae* | Tritimovirus | NP74274 | NP046741 |
| Sindbis virus |  | *Togaviridae* | Alphavirus | NP740673 | NP062888 |
| Rubella virus |  | *Togaviridae* | Rubivirus | NP740662 | NP062883 |
| Tobacco necrosis virus A |  | *Tombusviridae* | Alphanecrovirus | NP056828 | NP056825 |
| Pothos latent virus |  | *Tombusviridae* | Aureusvirus | YP009032636 | YP009032634 |
| Oat chlorotic stunt virus |  | *Tombusviridae* | Avenavirus | NP619753 | NP619751 |
| Tobacco necrosis virus D |  | *Tombusviridae* | Betanecrovirus | NP608316 | NP608311 |
| Carnation mottle virus |  | *Tombusviridae* | Carmovirus | YP009032648 | YP009032644 |
| Carnation ringspot virus |  | *Tombusviridae* | Dianthovirus | NP613255 | NP619711 |
| Galinsoga mosaic virus |  | *Tombusviridae* | Gallantivirus | NP044736 | NP044732 |
| Furcraea necrotic streak virus |  | *Tombusviridae* | Macanavirus | YP007517178 | YP007517174 |
| Maize chlorotic mottle virus |  | *Tombusviridae* | Machlomovirus | NP619722 | NP619718 |
| Panicum mosaic virus |  | *Tombusviridae* | Panicovirus | NP068346 | NP068342 |
| Tomato bushy stunt virus |  | *Tombusviridae* | Tombusvirus | NP062899 | NP062897 |
| Maize necrotic streak virus |  | *Tombusviridae* | Zeavirus | YP459922 | YP459920 |
| Rasberry bushy dwarf virus |  |  | Idaeovirus | NP620467 | NP624465 |
| Poinsettia latent virus |  |  | Polemovirus | YP002308463 | YP002308462 |
| Southern cowpea mosaic virus |  |  | Sobemovirus | NP042303 | NP042302 |
| Carrot mottle virus |  |  | Umbravirus | No capsid | YP002302259 |
| Soil-borne wheat mosaic virus |  | *Virgaviridae* | Furovirus | NP049338 | NP049335 |
| Barley stripe mosaic virus |  | *Virgaviridae* | Hordeivirus | NP604486 | NP604481 |
| Indian peanut clump virus |  | *Virgaviridae* | Pecluvirus | NP835263 | NP835282 |
| Potato mop-top virus |  | *Virgaviridae* | Pomovirus | NP620437 | NP620436 |
| Tobacco mosaic virus |  | *Virgaviridae* | Tobamovirus | NP597750 | NP597746 |
| Tobacco rattle virus |  | *Virgaviridae* | Tobravirus | NP620682 | NP620669 |

Blank entries indicate unassigned order or family.

## Additional Table 3: List of 223 genomic sequences from capsid sequences used for creating Additional Fig 2.

| **Accession number** | **Organism** | **Taxonomy ( where known: Order/Family/Genus)** |
| --- | --- | --- |
| NC_002470 | Turkey astrovirus | Astroviridae; Avastrovirus |
| NC_003790 | Chicken astrovirus | Astroviridae; Avastrovirus |
| NC_005790 | Turkey astrovirus 2 | Astroviridae; Avastrovirus |
| NC_012437 | Duck astrovirus C-NGB | Astroviridae; Avastrovirus |
| NC_013443 | HMO Astrovirus A | Astroviridae; Mamastrovirus |
| NC_016896 | Astrovirus wild boar/WBAstV-1/2011/HUN | Astroviridae; Mamastrovirus |
| NC_019494 | Porcine astrovirus 3 | Astroviridae; Mamastrovirus |
| NC_022249 | Feline astrovirus 2 | Astroviridae; Mamastrovirus |
| NC_023629 | Bovine astrovirus B76/HK | Astroviridae; Mamastrovirus |
| NC_023630 | Bovine astrovirus B76-2/HK | Astroviridae; Mamastrovirus |
| NC_023631 | Bovine astrovirus B18/HK | Astroviridae; Mamastrovirus |
| NC_023632 | Bovine astrovirus B170/HK | Astroviridae; Mamastrovirus |
| NC_023674 | Porcine astrovirus 2 | Astroviridae; Mamastrovirus |
| NC_023675 | Porcine astrovirus 4 | Astroviridae; Mamastrovirus |
| NC_024297 | Bovine astrovirus | Astroviridae; Mamastrovirus |
| NC_024472 | Human astrovirus BF34 | Astroviridae; Mamastrovirus |
| NC_024701 | Feline astrovirus D1 | Astroviridae; Mamastrovirus |
| NC_025346 | Rabbit astrovirus TN/2208/2010 | Astroviridae; Mamastrovirus |
| NC_026814 | Canine astrovirus | Astroviridae; Mamastrovirus |
| NC_027711 | Dromedary astrovirus | Astroviridae; Mamastrovirus |
| NC_011400 | Astrovirus MLB1 | Astroviridae; unclassified |
| NC_013060 | Astrovirus VA1 | Astroviridae; unclassified |
| NC_014320 | Astrovirus MLB1 HK05 | Astroviridae; unclassified |
| NC_015935 | Mouse astrovirus M-52/USA/2008 | Astroviridae; unclassified |
| NC_016155 | Astrovirus MLB2 | Astroviridae; unclassified |
| NC_018669 | Astrovirus VA2 | Astroviridae; unclassified |
| NC_018702 | Murine astrovirus | Astroviridae; unclassified |
| NC_019026 | Astrovirus VA3 | Astroviridae; unclassified |
| NC_019027 | Astrovirus VA4 | Astroviridae; unclassified |
| NC_019028 | Astrovirus MLB3 | Astroviridae; unclassified |
| NC_023636 | Porcine astrovirus 5 | Astroviridae; unclassified |
| NC_024498 | Bovine astrovirus CH13 | Astroviridae; unclassified |
| NC_025409 | Astrovirus SG | Astroviridae; unclassified |
| NC_027426 | Astrovirus Er/SZAL6/HUN/2011 | Astroviridae; unclassified |
| NC_003503 | Beet soil-borne mosaic virus | Benyviridae; Benyvirus |
| NC_003515 | Beet necrotic yellow vein virus | Benyviridae; Benyvirus |
| NC_021736 | Burdock mottle virus | Benyviridae; Benyvirus |
| NC_002025 | Alfalfa mosaic virus | Bromoviridae; Alfamovirus |
| NC_003651 | Pelargonium zonate spot virus | Bromoviridae; Anulavirus |
| NC_018404 | Amazon lily mild mottle virus | Bromoviridae; Anulavirus |
| NC_025484 | Cassava Ivorian bacilliform virus | Bromoviridae; Anulavirus |
| NC_002028 | Brome mosaic virus | Bromoviridae; Bromovirus |
| NC_003542 | Cowpea chlorotic mottle virus | Bromoviridae; Bromovirus |
| NC_004006 | Broad bean mottle virus | Bromoviridae; Bromovirus |
| NC_004122 | Spring beauty latent virus | Bromoviridae; Bromovirus |
| NC_007001 | Cassia yellow blotch virus | Bromoviridae; Bromovirus |
| NC_013268 | Melandrium yellow fleck virus | Bromoviridae; Bromovirus |
| NC_001440 | Cucumber mosaic virus | Bromoviridae; Cucumovirus |
| NC_002040 | Peanut stunt virus | Bromoviridae; Cucumovirus |
| NC_003836 | Tomato aspermy virus | Bromoviridae; Cucumovirus |
| NC_012136 | Gayfeather mild mottle virus | Bromoviridae; Cucumovirus |
| NC_003453 | American plum line pattern virus | Bromoviridae; Ilarvirus |
| NC_003480 | Apple mosaic virus | Bromoviridae; Ilarvirus |
| NC_003546 | Citrus leaf rugose virus | Bromoviridae; Ilarvirus |
| NC_003570 | Elm mottle virus | Bromoviridae; Ilarvirus |
| NC_003810 | Spinach latent virus | Bromoviridae; Ilarvirus |
| NC_003835 | Tulare apple mosaic virus | Bromoviridae; Ilarvirus |
| NC_003845 | Tobacco streak virus | Bromoviridae; Ilarvirus |
| NC_004364 | Prunus necrotic ringspot virus | Bromoviridae; Ilarvirus |
| NC_005854 | Parietaria mottle virus | Bromoviridae; Ilarvirus |
| NC_006066 | Humulus japonicus latent virus | Bromoviridae; Ilarvirus |
| NC_006568 | Fragaria chiloensis latent virus | Bromoviridae; Ilarvirus |
| NC_008038 | Prune dwarf virus | Bromoviridae; Ilarvirus |
| NC_008706 | Strawberry necrotic shock virus | Bromoviridae; Ilarvirus |
| NC_009536 | Citrus variegation virus | Bromoviridae; Ilarvirus |
| NC_011555 | Blackberry chlorotic ringspot virus | Bromoviridae; Ilarvirus |
| NC_011807 | Asparagus virus 2 | Bromoviridae; Ilarvirus |
| NC_022129 | Ageratum latent virus | Bromoviridae; Ilarvirus |
| NC_022252 | Blueberry shock virus | Bromoviridae; Ilarvirus |
| NC_025481 | Lilac leaf chlorosis virus | Bromoviridae; Ilarvirus |
| NC_027930 | Privet ringspot virus | Bromoviridae; Ilarvirus |
| NC_003671 | Olive latent virus 2 | Bromoviridae; Oleavirus |
| NC_001543 | Rabbit Haemorrhagic disease virus | Caliciviridae; Lagovirus |
| NC_007916 | Newbury agent 1 | Caliciviridae; Nebovirus |
| NC_001959 | Norwalk virus | Caliciviridae; Norovirus |
| NC_006269 | Sapovirus | Caliciviridae; Sapovirus |
| AB073912 | Swine hepatitis E virus | Hepeviridae; Orthohepevirus |
| AB074915 | Hepatitis E virus | Hepeviridae; Orthohepevirus |
| AB074917 | Hepatitis E virus | Hepeviridae; Orthohepevirus |
| AB074918 | Hepatitis E virus | Hepeviridae; Orthohepevirus |
| AB074920 | Hepatitis E virus | Hepeviridae; Orthohepevirus |
| AB080575 | Hepatitis E virus | Hepeviridae; Orthohepevirus |
| AB089824 | Hepatitis E virus | Hepeviridae; Orthohepevirus |
| AB091394 | Hepatitis E virus | Hepeviridae; Orthohepevirus |
| AB097811 | Swine hepatitis E virus | Hepeviridae; Orthohepevirus |
| AB097812 | Hepatitis E virus | Hepeviridae; Orthohepevirus |
| AB740220 | Rabbit hepatitis E virus | Hepeviridae; Orthohepevirus |
| AB740221 | Rabbit hepatitis E virus | Hepeviridae; Orthohepevirus |
| AB740222 | Rabbit hepatitis E virus | Hepeviridae; Orthohepevirus |
| AB847305 | Hepatitis E virus | Hepeviridae; Orthohepevirus |
| AB847306 | Hepatitis E virus | Hepeviridae; Orthohepevirus |
| AB847307 | Hepatitis E virus | Hepeviridae; Orthohepevirus |
| AB847308 | Hepatitis E virus | Hepeviridae; Orthohepevirus |
| AB847309 | Hepatitis E virus | Hepeviridae; Orthohepevirus |
| AB890001 | Ferret hepatitis E virus | Hepeviridae; Orthohepevirus |
| AB890374 | Ferret hepatitis E virus | Hepeviridae; Orthohepevirus |
| D11092 | Hepatitis E virus | Hepeviridae; Orthohepevirus |
| FJ906895 | Rabbit hepatitis E virus | Hepeviridae; Orthohepevirus |
| FJ906896 | Rabbit hepatitis E virus | Hepeviridae; Orthohepevirus |
| GU345042 | Rat hepatitis E virus | Hepeviridae; Orthohepevirus |
| GU345043 | Rat hepatitis E virus | Hepeviridae; Orthohepevirus |
| GU937805 | Rabbit hepatitis E virus | Hepeviridae; Orthohepevirus |
| JF443717 | Hepatitis E virus | Hepeviridae; Orthohepevirus |
| JF443718 | Hepatitis E virus | Hepeviridae; Orthohepevirus |
| JF443719 | Hepatitis E virus | Hepeviridae; Orthohepevirus |
| JN167537 | Hepatitis E virus | Hepeviridae; Orthohepevirus |
| JN167538 | Hepatitis E virus | Hepeviridae; Orthohepevirus |
| JN998606 | Ferret hepatitis E virus | Hepeviridae; Orthohepevirus |
| JN998607 | Ferret hepatitis E virus | Hepeviridae; Orthohepevirus |
| JQ013791 | Rabbit hepatitis E virus | Hepeviridae; Orthohepevirus |
| JQ013792 | Rabbit hepatitis E virus | Hepeviridae; Orthohepevirus |
| JQ768461 | Rabbit hepatitis E virus | Hepeviridae; Orthohepevirus |
| JX109834 | Rabbit hepatitis E virus | Hepeviridae; Orthohepevirus |
| JX120573 | Hepatitis E virus | Hepeviridae; Orthohepevirus |
| JX121233 | Rabbit hepatitis E virus | Hepeviridae; Orthohepevirus |
| JX565469 | Rabbit hepatitis E virus | Hepeviridae; Orthohepevirus |
| KJ013414 | Rabbit hepatitis E virus | Hepeviridae; Orthohepevirus |
| KJ013415 | Rabbit hepatitis E virus | Hepeviridae; Orthohepevirus |
| KJ496143 | Camel hepatitis E virus | Hepeviridae; Orthohepevirus |
| KJ496144 | Camel hepatitis E virus | Hepeviridae; Orthohepevirus |
| KJ507955 | Swine hepatitis E virus | Hepeviridae; Orthohepevirus |
| KJ507956 | Swine hepatitis E virus | Hepeviridae; Orthohepevirus |
| KJ701409 | Hepatitis E virus | Hepeviridae; Orthohepevirus |
| KM516906 | Hepatitis E virus | Hepeviridae; Orthohepevirus |
| L25595 | Hepatitis E virus | Hepeviridae; Orthohepevirus |
| M73218 | Hepatitis E virus | Hepeviridae; Orthohepevirus |
| M74506 | Hepatitis E virus | Hepeviridae; Orthohepevirus |
| M80581 | Hepatitis E virus | Hepeviridae; Orthohepevirus |
| M94177 | Hepatitis E virus | Hepeviridae; Orthohepevirus |
| NC_001434 | Hepatitis E virus | Hepeviridae; Orthohepevirus |
| X98292 | Hepatitis E virus | Hepeviridae; Orthohepevirus |
| X99441 | Hepatitis E virus | Hepeviridae; Orthohepevirus |
| NC_015521 | Cutthroat trout virus | Hepeviridae; Piscihepevirus |
| AM943646 | Avian hepatitis E virus | Hepeviridae; Orthohepevirus |
| AM943647 | Avian hepatitis E virus | Hepeviridae; Orthohepevirus |
| EF206691 | Avian hepatitis E virus | Hepeviridae; Orthohepevirus |
| GU954430 | Avian hepatitis E virus | Hepeviridae; Orthohepevirus |
| JN597006 | Avian hepatitis E virus | Hepeviridae; Orthohepevirus |
| JN997392 | Avian hepatitis E virus | Hepeviridae; Orthohepevirus |
| KC454286 | Avian hepatitis E virus | Hepeviridae; Orthohepevirus |
| KF511797 | Avian hepatitis E virus | Hepeviridae; Orthohepevirus |
| NC_023425 | Avian hepatitis E virus | Hepeviridae; Orthohepevirus |
| JQ001749 | Bat hepevirus | Hepeviridae; Orthohepevirus |
| KJ562187 | Bat hepevirus | Hepeviridae; Orthohepevirus |
| NC_018382 | Bat hepevirus | Hepeviridae; Orthohepevirus |
| NC_005985 | Pelargonium chlorotic ring pattern virus | Tombusviridae |
| NC_007017 | Pelargonium line pattern virus | Tombusviridae |
| NC_015227 | Trailing lespedeza virus 1 | Tombusviridae |
| NC_020415 | Rosa rugosa leaf distortion virus | Tombusviridae |
| NC_026240 | Pelargonium ringspot virus | Tombusviridae |
| NC_001721 | Olive latent virus 1 | Tombusviridae; Alphanecrovirus |
| NC_001777 | Tobacco necrosis virus A | Tombusviridae; Alphanecrovirus |
| NC_006939 | Olive mild mosaic virus | Tombusviridae; Alphanecrovirus |
| NC_000939 | Pothos latent virus | Tombusviridae; Aureusvirus |
| NC_005287 | Johnson grass chlorotic stripe mosaic virus | Tombusviridae; Aureusvirus |
| NC_007816 | Cucumber leaf spot virus | Tombusviridae; Aureusvirus |
| NC_009533 | Maize white line mosaic virus | Tombusviridae; Aureusvirus |
| NC_022895 | Yam spherical virus | Tombusviridae; Aureusvirus |
| NC_003633 | Oat chlorotic stunt virus | Tombusviridae; Avenavirus |
| NC_003487 | Tobacco necrosis virus D | Tombusviridae; Betanecrovirus |
| NC_004452 | Beet black scorch virus | Tombusviridae; Betanecrovirus |
| NC_001265 | Carnation mottle virus | Tombusviridae; Carmovirus |
| NC_001600 | Cardamine chlorotic fleck virus | Tombusviridae; Carmovirus |
| NC_002187 | Japanese iris necrotic ring virus | Tombusviridae; Carmovirus |
| NC_003535 | Cowpea mottle virus | Tombusviridae; Carmovirus |
| NC_003608 | Hibiscus chlorotic ringspot virus | Tombusviridae; Carmovirus |
| NC_003821 | Turnip crinkle virus | Tombusviridae; Carmovirus |
| NC_005286 | Pelargonium flower break virus | Tombusviridae; Carmovirus |
| NC_007733 | Angelonia flower break virus | Tombusviridae; Carmovirus |
| NC_009017 | Nootka lupine vein clearing virus | Tombusviridae; Carmovirus |
| NC_011643 | Soybean yellow mottle mosaic virus | Tombusviridae; Carmovirus |
| NC_014967 | Honeysuckle ringspot virus | Tombusviridae; Carmovirus |
| NC_021926 | Calibrachoa mottle virus | Tombusviridae; Carmovirus |
| NC_026239 | Elderberry latent virus | Tombusviridae; Carmovirus |
| NC_003530 | Carnation ringspot virus | Tombusviridae; Dianthovirus |
| NC_003756 | Red clover necrotic mosaic virus | Tombusviridae; Dianthovirus |
| NC_001818 | Galinsoga mosaic virus | Tombusviridae; Gallantivirus |
| NC_020469 | Furcraea necrotic streak virus | Tombusviridae; Macanavirus |
| NC_003627 | Maize chlorotic mottle virus | Tombusviridae; Machlomovirus |
| NC_002598 | Panicum mosaic virus | Tombusviridae; Panicovirus |
| NC_011108 | Cocksfoot mild mosaic virus | Tombusviridae; Panicovirus |
| NC_021705 | Thin paspalum asymptomatic virus | Tombusviridae; Panicovirus |
| NC_001339 | Artichoke mottled crinkle virus | Tombusviridae; Tombusvirus |
| NC_001469 | Cucumber necrosis virus | Tombusviridae; Tombusvirus |
| NC_001554 | Tomato bushy stunt virus | Tombusviridae; Tombusvirus |
| NC_003500 | Carnation Italian ringspot virus | Tombusviridae; Tombusvirus |
| NC_003532 | Cymbidium ringspot virus | Tombusviridae; Tombusvirus |
| NC_004725 | Cucumber Bulgarian virus | Tombusviridae; Tombusvirus |
| NC_005285 | Pelargonium necrotic spot virus | Tombusviridae; Tombusvirus |
| NC_011535 | Grapevine Algerian latent virus | Tombusviridae; Tombusvirus |
| NC_020073 | Moroccan pepper virus | Tombusviridae; Tombusvirus |
| NC_023339 | Eggplant mottled crinkle virus | Tombusviridae; Tombusvirus |
| NC_007729 | Maize necrotic streak virus | Tombusviridae; Zeavirus |
| NC_003347 | Grapevine fleck virus | Tymovirales; Tymoviridae; Maculavirus |
| NC_015524 | Bombyx mori Macula-like virus | Tymovirales; Tymoviridae; Maculavirus |
| NC_018703 | Culex originated Tymoviridae-like virus | Tymovirales; Tymoviridae; Maculavirus |
| NC_001793 | Oat blue dwarf virus | Tymovirales; Tymoviridae; Marafivirus |
| NC_002786 | Maize rayado fino virus | Tymovirales; Tymoviridae; Marafivirus |
| NC_006950 | Citrus sudden death-associated virus | Tymovirales; Tymoviridae; Marafivirus |
| NC_012484 | Grapevine Syrah virus 1 | Tymovirales; Tymoviridae; Marafivirus |
| NC_013920 | Olive latent virus 3 | Tymovirales; Tymoviridae; Marafivirus |
| NC_001480 | Eggplant mosaic virus | Tymovirales; Tymoviridae; Tymovirus |
| NC_001513 | Ononis yellow mosaic virus | Tymovirales; Tymoviridae; Tymovirus |
| NC_001746 | Kennedya yellow mosaic virus | Tymovirales; Tymoviridae; Tymovirus |
| NC_001977 | Erysimum latent virus | Tymovirales; Tymoviridae; Tymovirus |
| NC_002588 | Chayote mosaic virus | Tymovirales; Tymoviridae; Tymovirus |
| NC_003634 | Physalis mottle virus | Tymovirales; Tymoviridae; Tymovirus |
| NC_004063 | Turnip yellow mosaic virus | Tymovirales; Tymoviridae; Tymovirus |
| NC_007609 | Dulcamara mottle virus | Tymovirales; Tymoviridae; Tymovirus |
| NC_009532 | Okra mosaic virus | Tymovirales; Tymoviridae; Tymovirus |
| NC_011086 | Diascia yellow mottle virus | Tymovirales; Tymoviridae; Tymovirus |
| NC_011537 | Scrophularia mottle virus | Tymovirales; Tymoviridae; Tymovirus |
| NC_011538 | Nemesia ring necrosis virus | Tymovirales; Tymoviridae; Tymovirus |
| NC_011539 | Plantago mottle virus | Tymovirales; Tymoviridae; Tymovirus |
| NC_011559 | Anagyris vein yellowing virus | Tymovirales; Tymoviridae; Tymovirus |
| NC_014127 | Chiltepin yellow mosaic virus | Tymovirales; Tymoviridae; Tymovirus |
| NC_015523 | Asclepias asymptomatic virus | Tymovirales; Tymoviridae; Tymovirus |
| NC_020470 | Andean potato latent virus | Tymovirales; Tymoviridae; Tymovirus |
| NC_020471 | Andean potato mild mosaic virus | Tymovirales; Tymoviridae; Tymovirus |
| NC_021851 | Tomato blistering mosaic virus | Tymovirales; Tymoviridae; Tymovirus |
| NC_002164 | Poinsettia mosaic virus | Tymovirales; Tymoviridae; unassigned |
| NC_027619 | Varroa Tymo-like virus | Tymovirales; Tymoviridae; unclassified |
| NC_027631 | Bee Macula-like virus | Tymovirales; Tymoviridae; unclassified |
